# Gut microbiome-mediated metabolism effects on immunity in rural and urban African populations

**DOI:** 10.1038/s41467-021-25213-2

**Published:** 2021-08-11

**Authors:** Martin Stražar, Godfrey S. Temba, Hera Vlamakis, Vesla I. Kullaya, Furaha Lyamuya, Blandina T. Mmbaga, Leo A. B. Joosten, Andre J. A. M. van der Ven, Mihai G. Netea, Quirijn de Mast, Ramnik J. Xavier

**Affiliations:** 1 Broad Institute of MIT and Harvard, Cambridge, MA USA; 2 Kilimanjaro Christian Medical University College, Moshi, Tanzania; 3 Kilimanjaro Clinical Research Institute, Kilimanjaro Christian Medical Centre, Moshi, Tanzania; 4 Department of Respiratory Medicine, Radboud University Medical Center, Nijmegen, The Netherlands; 5 Department of Pediatrics, Kilimanjaro Clinical Research Institute, Kilimanjaro Christian Medical Centre, Moshi, Tanzania; 6 Department of Internal Medicine, Radboud University Medical Center, Nijmegen, The Netherlands; 7 Center for Infectious Diseases, Radboud University Medical Center, Nijmegen, The Netherlands; 8 Center for Microbiome Informatics and Therapeutics, Massachusetts Institute of Technology, Cambridge, MA USA; 9 Center for Computational and Integrative Biology and Department of Molecular Biology, Massachusetts General Hospital and Harvard Medical School, Boston, MA USA

**Keywords:** Cytokines, Bacteria

## Abstract

The human gut microbiota is increasingly recognized as an important factor in modulating innate and adaptive immunity through release of ligands and metabolites that translocate into circulation. Urbanizing African populations harbor large intestinal diversity due to a range of lifestyles, providing the necessary variation to gauge immunomodulatory factors. Here, we uncover a gradient of intestinal microbial compositions from rural through urban Tanzanian, towards European samples, manifested both in relative abundance and genomic variation observed in stool metagenomics. The rural population shows increased *Bacteroidetes*, led by *Prevotella copri*, but also presence of fungi. Measured ex vivo cytokine responses were significantly associated with 34 immunomodulatory microbes, which have a larger impact on circulating metabolites than non-significant microbes. Pathway effects on cytokines, notably TNF-α and IFN-γ, differential metabolome analysis and enzyme copy number enrichment converge on histidine and arginine metabolism as potential immunomodulatory pathways mediated by *Bifidobacterium longum* and *Akkermansia muciniphila*.

## Abstract (web summary)

The authors profile stool metagenomics and plasma metabolomics in Tanzanian individuals and uncover a gradient of gut microbial profiles, from rural through urban Tanzania towards Western populations. Integration with ex vivo blood microbial stimulations reveals immune responses associated with histidine and arginine pathways, mediated by *Bifidobacterium longum* and *Akkermansia muciniphila*.

## Introduction

The complex relationship between humans and commensal gut microbes is vital to maintaining intestinal homeostasis, and disruption of this symbiosis can drive inflammation and development of inflammatory disease. Lifestyle and behavioral changes resulting from unprecedented economic development and migration are major contributors to shifts in the intestinal ecosystem^1–5^. These environmental changes exert a more rapid impact than host genetic determinants of microbiome composition^6^, and are increasingly attributed to a rise in autoimmune diseases including rheumatoid arthritis, multiple sclerosis, type 1 diabetes, or inflammatory bowel disease. The urbanization gradient, which refers to the variation of environmental factors with migration and development, has wide-ranging impacts on factors that affect host-microbiome interactions and overall health. These include diet and healthcare access, as well as early life factors such as delivery mode, breastfeeding, and antibiotic use that affect neonatal microbial colonization^7^. Large population studies indicate that the full landscape of microbial diversity is still underexplored^3^, particularly in industrializing countries^1–3^.

The host immune system and the microbiota interface through microbial ligands or metabolites^8,9^, which regulate gut permeability, mucus layer integrity, or polarization of T cells to their subtypes^10^. The latter can be induced ex vivo by common gut colonizers including *E. coli*^11^, *S. aureus*^12^, or *C. albicans*^13^, inducing detectable changes in monocyte- and lymphocyte-produced cytokine expression. Studies in humans^14^ and antibiotic-treated and germ-free mice^15,16^ identified microbial stimuli and vaccines that modulate immune responses. For example, functional metagenomic studies in cohorts of healthy individuals of Western European ethnicity identified associations between expression of host pro-inflammatory cytokines and microbial tryptophan and palmitoleic acid metabolic pathways^17^. Training of the innate immune system in childhood can be affected by differences in structures of lipopolysaccharide cell wall components from *E. coli* and *Bacteroides*^18^. Alterations in microbial metabolite pools (acylcarnitines, bile acids, and short-chain fatty acids) can also predict inflammatory bowel disease types^19,20^. However, the molecular mechanisms and metabolites through which microbial species affect cytokine responses in vivo remain unknown.

Identifying precise immunomodulatory pathways has been challenging due to limited computational tools and reference genomes^21^, requiring cohorts harboring large microbial diversity. African populations encompass a diversity of lifestyles and opportunities to detect ecological niches^5,22,23^, as well as a pervasiveness of autoimmune diseases^24^ and epidemics^25,26^. Leveraging the diversity of these populations to dissect the dynamics of competitive microbial interactions that have led to the evolution of complex molecules that allow bacteria to interact with host immunity and utilize it to their own advantage^27–29^ might unveil promising therapeutic strategies or dietary interventions.

Here we present a Tanzanian cohort with paired stool metagenomics, plasma metabolomics, and ex vivo blood stimulations with eight microbial stimuli, covering a range of immune system modalities, and we compare it with a cohort of Dutch volunteers. Subjects from the Tanzanian cohort hail from both urban and rural settings, representing a range of lifestyles, diet, and microbial exposures that reveal a gradient of microbial compositions. The consequent variation in cytokine responses, most notably TNF-α and IFN-γ, is linked to 34 immunomodulatory microbes with increased effects on independently measured circulating metabolites. Multiple lines of evidence converge to histidine and arginine metabolism as microbially-mediated immunomodulatory pathways.

## Results

### Tanzanian gut microbial compositions diverge from Western populations along the urbanization gradient

**Fig. 1:**
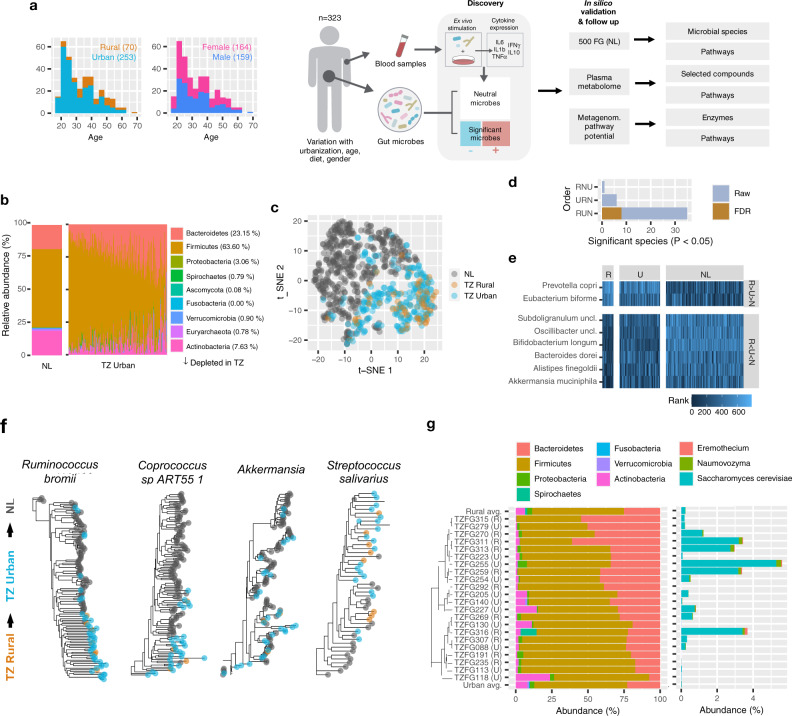
Microbial and immune system differences change across the Western-Urban-Rural gradient. **a** Study design overview. Stool metagenomics, plasma metabolomics, and ex vivo blood stimulation were collected for all study participants (*n* = 323). The microbes are first tested for significant effects on inducing differential cytokine expression. The resulting immunomodulatory species are compared against the NL cohort and plasma metabolome of the original TZ samples. Furthermore, immunomodulatory species are compared to non-significant species for differences in encoded metabolomic pathways using the same stool metagenomics data. **b** Relative abundance of phyla. Average in the NL (left), Tanzanian urban samples (center), and Tanzanian rural samples (right). **c** A t-SNE plot based on Jensen-Shannon distance, comparing Tanzanian (TZ) relative species abundance profiles with 500 FG cohort from the Netherlands (NL). The two cohorts had 178 microbial species in common (out of 282 Tanzania and 219 Netherlands). **d** Number of detected species simultaneously changing by all possible gradients: rural-to-urban-to-Netherlands (RUN), urban-to-rural-to-Netherlands (URN), or rural-to-Netherlands-to-Urban (RNU). Bars show the number of significant species from the double Wilcoxon rank-sum test (see “Methods”), uncorrected or FDR-corrected. **e** Species simultaneously changing along the TZ rural versus urban and TZ urban versus NL. Displayed are ranked values, samples with no detected species have rank zero. **f** Selected species diverging in NL, TZ urban, and TZ rural (StrainPhlan). **g** Twenty-two samples with detected fungi, along with phylum relative abundance. Source data are provided in the Source data file.

The presented Tanzanian (TZ) cohort consists of 70 subjects from rural (median age 39.6) and 253 from urban areas (TZ, median age 27.6), with a well-balanced gender distribution (Fig. 1a). A participant survey comprised 54 anthropometric and lifestyle-related variables, including diet, antibiotics courses, animal exposure, pollution, and quality of drinking water (Supplementary Data 1-2). The individuals were subjected to stool metagenomic sequencing, plasma metabolomic profiling, and ex vivo stimulation of whole blood with a panel of well-studied microbes.

Reference-based stool metagenomics revealed 415 species from 154 genera (Metaphlan^30^). Only 212 species were present in at least 10 individuals, implying that approximately half of the species are rare and highly specific. A previously published cohort of 471 individuals from the Netherlands (NL) detected 407 species from 134 genera, yielding less taxa despite having 1.5 times more samples^17^. Both TZ and NL cohorts were profiled using marker gene-based method Metaphlan and showed a comparable number of reads mapping to both marker genes and UniRef90 gene families (Supplementary Fig. 1a-c). As the TZ cohort yielded more reads on average, the smaller proportion of reads mapping to marker genes may be attributed both to presence of yet uncatalogued taxa and difference in sequencing protocols.

Relative abundances of shared phyla differentiated TZ and NL individuals (Fig. 1b). This manifested through a reduction in *Actinobacteria* (7.6% in TZ vs. 18.3% in NL, *P*_FDR_ = 9.39E−39) and an increase in *Bacteroidetes* (23.1% vs. 18.2%, *P*_FDR_ = 2.30E−08), *Firmicutes* (63.6% vs. 58.7%, *P*_FDR_ < 1.12E−05) and *Proteobacteria* (3.1% vs. 0.5%, *P*_FDR_ = 1.30E−36). Residency was differentiated further by *Actinobacteria* (6.3% rural vs. 8.1% urban, *P*_FDR_ =0.241) and *Verrucomicrobia* (0.2% vs. 1.1%, *P*_FDR_ = 0.241). *Verrucomicrobia* is rare in traditional populations^1^ and their 5.5-fold increase in urban TZ suggests a continuum of gut compositions ordered from rural, through urban Tanzania, and toward Western Europe.

Seeking confirmation of the manifested urbanization gradient, we profiled samples using 176 shared species between the two cohorts which showed comparable detection rates in the three subgroups (Supplementary Fig. 1d). A t-SNE plot based on species profiles confirmed cohort divergence, with rural samples in particular showing strong polarization (Fig. 1c). The observed projection was in turn not visibly driven by age, sex, or number of marker genes (Supplementary Fig. 1e). We performed two Wilcoxon rank-sum tests for each species to find simultaneous changes in abundance between (1) rural and urban and (2) urban and NL samples. This revealed eight species changing along the gradient (Fig. 1e, Supplementary Data 3, *P*_FDR_ < 0.05, see the “Methods” section). The two alternative orderings of tests (urban-to-rural-to-NL or rural-to-NL-to-urban) did not yield any associations (Fig. 1d). While sex and age expectedly had significant effects on the microbial compositions (explained variance 2.3% and 1.7%, respectively, PERMANOVA, Supplementary Fig. 1f), the rural-to-urban-to-NL had a comparably stronger effect (explained variance 16.6%, Supplementary Fig. 1f), increasing our confidence in the perceived rural-to-urban-to-NL gradient.

Species more abundant in rural populations were *Eubacterium biforme* and highly abundant *Prevotella copri*, which is characteristic for African populations^22^, in particular hunters^1^. Six species increased toward the NL population, including *Akkermansia muciniphila* and *Bifidobacterium longum*, the latter 97% prevalent in NL but only 61% in TZ (Supplementary Fig. 2, Supplementary Data 4). Another characteristic Firmicute for NL was *Subdoligranulum*, which is related to *Faecalibacterium prausnitzii*^31^. To our surprise, a comparison between entire cohorts (NL versus TZ, without urban/rural separation) yielded *F. prausnitzii* as the strongest from 43 enriched species in TZ, suggesting that phylogenetically similar microbes can show reverse abundance trends in geographically distant locations.

Functional differences resulting from differential abundance were supported by pathway profiling using Humann2. Twenty-one MetaCyc pathways showed differential copy numbers, sixteen of which were enriched in TZ (Supplementary Fig. 3). These were dominated by branched-chain amino acids valine and isoleucine biosynthesis, previously observed to be active in *P. copri* and associated with insulin resistance^32^. The Dutch samples showed an enrichment of lactose and galactose degradation, potentially reflecting different dietary habits.

Potential adaptations below species level were investigated with phylogenetic strain analysis (Fig. 1f; Supplementary Fig. 4; Supplementary Data 5; “Methods”). Seventeen species and two genera showed significant strain-level divergence between rural, urban, and NL samples (PERMANOVA, *P* < 0.05). These include the aforementioned *Akkermansia*, differentially abundant *Ruminococcus bromii* and *Lactobacillus ruminis*, but also five taxa not detected with differential abundance (*P*_FDR_ > 0.05, Supplementary Data 5): *Ruminococcus obeum*, *Roseburia hominis*, *Clostridium sp L2 50*, *Streptococcus salivarius*, *Coprococcus sp ART55 1*. These genetic differences complement differential abundance and highlight an alternative route for functional effects to take place.

The rural microbiome traits can also be seen with eukaryotes, which are rarely detected but have large potential to affect microbial ecosystems^22^. Twenty-two samples contained at least one of three detected fungal taxa: *Saccharomyces cerevisiae*, *Naumovozyma*, and *Eremothecium*, the latter two only classified at the genus level (Fig. 1g). While half (11) of these cases were rural (*P* = 0.002, Chi-square test), the remaining 11 urban cases also showed strong rural resemblance. Seventeen out of 22 cases had less than half the expected Actinobacteria abundance (median 1.4% compared to average 6.3% in rural). Finally, since only 4 NL samples yielded any fungi, the mycobiome presents another understudied facet of gut diversity.

### Environmental, socio-economic, and dietary factors shape the gut microbiota

**Fig. 2:**
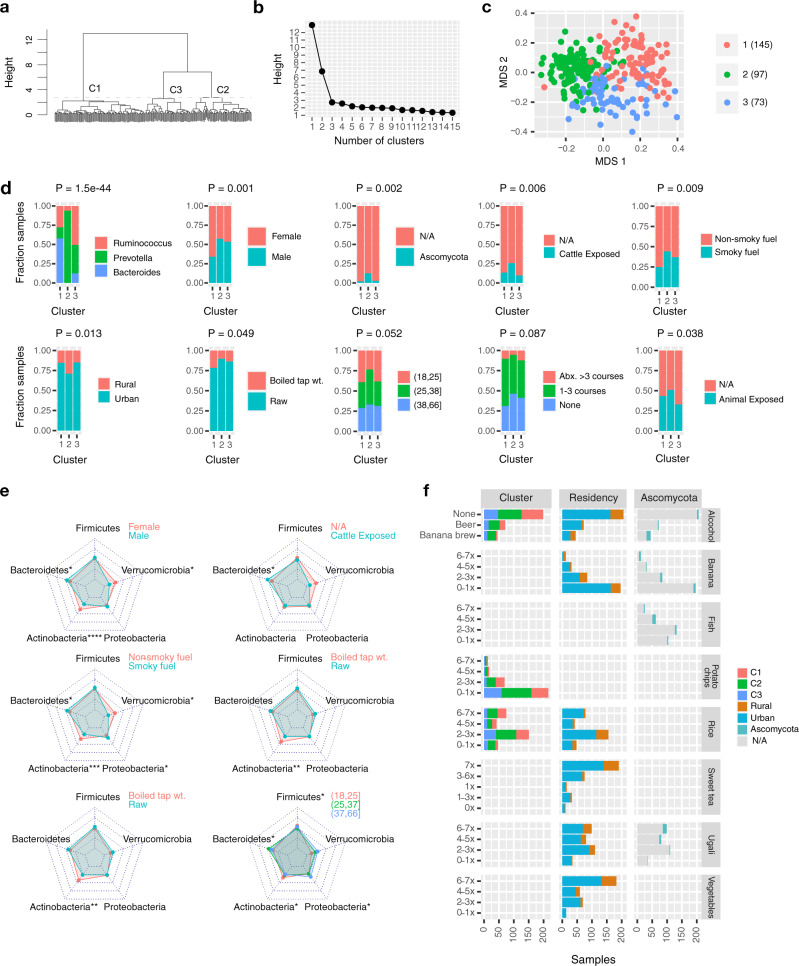
Environmental factors drive the Tanzanian microbial compositions. **a** Dendrogram of samples based on Jensen-Shannon distances between species relative abundances. **b** Three clusters are determined based on dendrogram height. **c** Multidimensional scaling (MDS) of cohort. **d** Cluster dependence on anthropometric variables (Chi-square test with unadjusted *P*-values <0.05), of which eight most associated are shown: enterotype, age interval, gender, Ascomycota presence, cattle exposure, fuel type, residency, and drinking water quality. **e** Changes in phyla abundance with selected variables using Maaslin2 (two-tailed t-test, *P*_FDR_ < 0.25). Radar plots span the 5 and 95% percentiles of relative abundances for each phylum (*P ≤ 0.05, **P ≤ 0.01, ***P ≤ 0.001, ****P ≤ 0.0001 for exact *P*-values). **f** Dietary factors significantly associated with microbiome clusters, residency, and presence of fungi (*P* < 0.05, Chi-square test or Kruskal–Wallis test, “Methods”). Ranges on the *y*-axis indicate weekly frequency or type of consumed food or drink. Source data are provided in the Source data file.

Differences in living standards and lifestyles across Tanzania determined great within-cohort microbial diversity. Species-level relative abundances were used to summarize Tanzanian metagenomes and revealed three main clusters driven by environmental and anthropometric factors (Fig. 2a–c, “Methods”). The largest (cluster 1) was dominated by *Bacteroides* and contained younger, urban females (Fig. 2d). The second cluster (2) revealed typical profiles from the non-urban areas: it contained the majority of rural samples (42/70), dominance of *Prevotella*, increased exposure to cattle, and smoky fuel. This group also included almost all the cases (18 out of 22) where *Ascomycota* were detected. The remaining cluster (3) contained urban males, was dominated by *Ruminococcus* and *Prevotella*, and was least exposed to animals.

Beyond clustering, the identified factors each significantly shifted the composition of at least one phylum. Women had a larger proportion of *Actinobacteria* and *Verrucomicrobia*, while men had an increase in *Bacteroidetes* (*P*_FDR_ < 0.25, Fig. 2e, Supplementary Data 6). Urbanization and presence of fungi affected these same three phyla, as presented above.

A dietary survey revealed how frequencies and types of consumed food and drinks differentiate between urban and rural samples, determine fungal presence and shape the clustering (Fig. 2f, Chi-square/Kruskal–Wallis test, *P* < 0.05, Supplementary Data 7). Urbanization was aligned with most dietary variables (six), where rural samples were increasingly likely to consume vegetables, ugali (maize porridge), and sweet tea. Alcohol intake was affecting all three aspects and revealed banana brew (a popular indigenous alcoholic beverage in the Kilimanjaro area made from fermented bananas and finger millet) as a potential cause of fungal gut colonization. Taken together, the detected environmental and dietary effects may both affect the microbiome or reflect the lifestyle differences to various degrees.

### Microbiome induces systematic trends in cytokine expression

**Fig. 3:**
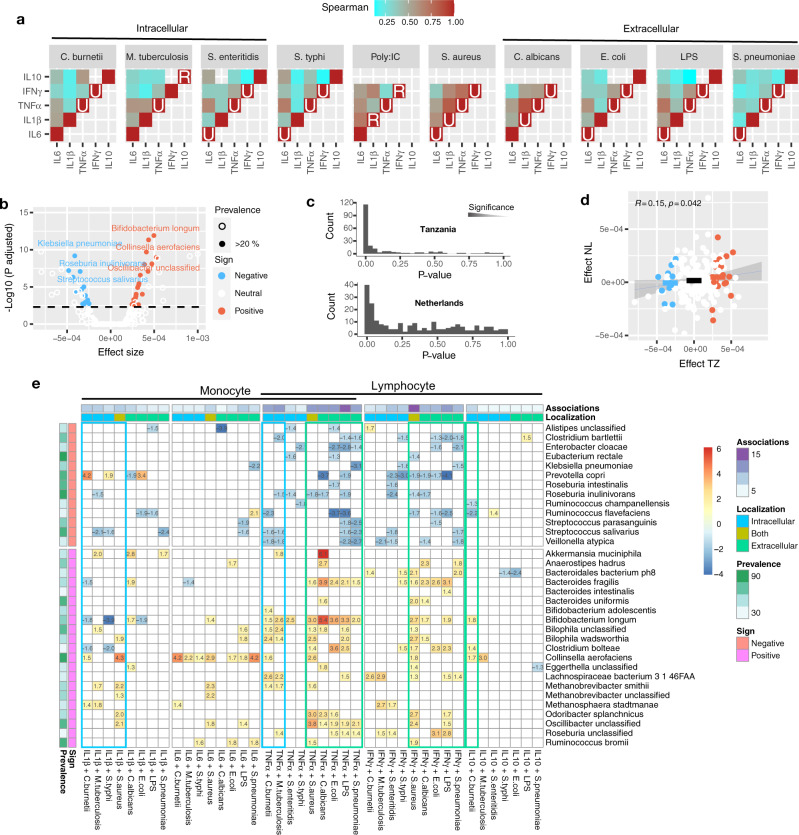
Identification of immunomodulatory species. **a** Whole blood from study participants was stimulated ex vivo with one fungal and seven bacterial stimuli and expression of five cytokines was measured. Heatmaps show significant Spearman correlations (*P* < 0.05) between samples in cytokine responses to all pairs of stimuli. Diagonal cells display significantly increased levels in urban (U) or rural (R) samples (Wilcoxon rank-sum two-sided test, *P* < 0.05). **b** Volcano plot of the species effects in cytokine responses, based on the log-linear model log *y*_p,m,i_ = *β*_0_ + *β*_d_dep_i_ + *β*_m_mic_m,i_ + *β*_e_eff_m_ + *ε*_p,m,i_, where *ε*_c,s,i_ is the noise, *β*_0_ the intercept, *β*_d_ the subject sequencing depth, *β*_m_ microbe abundance, and *β*_e_ the microbes’ immunomodulatory (“Methods”). Immunomodulatory species are further split by negative (13, blue) and positive (21, red) effects subject to *P*_Bonferroni_ < 0.005 (two-tailed t-test) and prevalence >20%. **c** Distribution of *p*-values for each species resulting from the log-linear model used in TZ (top) and NL (bottom) cohorts. **d** Comparison with the same log-linear model applied to intersecting ex vivo blood cell stimulation from the NL cohort. Macrophages were stimulated with LPS, *C. albicans*, *S. typhi*, and *M. tuberculosis*, and IL-6 and TNF-α were measured. Point colors refer to directionality of immunomodulatory species as in (**b**). **e** Individual effects of immunomodulatory microbes. Coefficients are obtained from a linear model log(Cytokine expression) ~ age + gender + microbe fit to each individual cytokine and stimulus combination and only significant values are shown (two-tailed t-test, unadjusted *P* < 0.05; see Supplementary Data 9 for exact *P*-values). Source data are provided in the Source data file.

Immunological profiles of Tanzanian subjects with different lifestyles and gut microbiota were assessed by cytokine responses of whole blood samples upon exposure to microbial and fungal stimuli. The analysis strategy to identify immunomodulatory microbes is outlined in Fig. 1a. Cytokine production^33^ in whole blood was stimulated by Gram-positive (*Staphylococcus aureus* and *Streptococcus pneumoniae*), Gram-negative bacteria (*Salmonella typhi*, *Coxiella burnetii*, and *Escherichia coli*), *Mycobacterium tuberculosis*, the yeast *Candida albicans*, as well as Toll-like receptor-3 and -4 ligands. The rural population had lower cytokine responses overall, as we also recently reported, (Fig. 3a, Supplementary Fig. 5a-b, Supplementary Data 8 and Temba et al.^33^), most notably in stimulation of TNF-α with LPS (*P* < 10^−4^, Wilcoxon rank-sum test), *S. aureus* (*P* < 10^−4^), and *C. albicans* (*P* < 10^−3^). Another notable cytokine was IFN-γ, eliciting stronger responses to intracellular compared to extracellular stimuli, consistent with its role in lymphocyte-induced macrophage activation. Delineation of whole blood leukocyte and differential counts prior to stimulation revealed positive correlation between monocytes and IL-6, TNF-α, IL-1β, and IL-10, while IFN-γ expression was more strongly associated with lymphocytes (Supplementary Fig. 5c). As the changes in cytokine levels may stem both from changes in blood cell composition or altered transcription, the proportional effect of each mechanism would require targeted experiments. The unstimulated monocytes and lymphocyte counts were however indistinguishable between rural and urban samples (Supplementary Fig. 5d), indicating that the main cohort stratification is unlikely to be confounded by cellular composition. Together, the variation in cytokine production suggests that environmental factors may affect the immune responses.

Cytokine production was significantly correlated between almost all cytokines and stimuli (71 out of 79 combinations, Fig. 3a, Supplementary Fig. 5b), except Poly:IC, suggesting that multiple cytokines can be combined to detect an immune response to a stimulus and its associations. We designed a log-linear model to test for a systematic increase in all combinations of cytokines and stimuli, conditioned on microbial abundances, age and sex (“Methods”). High cytokine expression levels were inversely associated with age (*P* < 10^−33^) and male gender (*P* = 0.014). Along with these factors, 34 stringently filtered species were identified (*P*_Bonferroni_ < 0.005, prevalence >20%, Fig. 3b; Supplementary Data 9), and split by positive (21) and negative (13) effects on ex vivo cytokine expression.

Concordant results were obtained with the same method on the NL cohort which had a similar experimental design including macrophage stimulations with overlap to the TZ study in four stimuli, IL-6 and TNF-α^17^ (Supplementary Fig. 6a). Despite fewer significant microbes from the NL cohort (Fig. 3c, Supplementary Fig. 6b), the effects were consistent on average (Fig. 3d; *P* = 0.042, Pearson correlation test). Differences may arise due to lower diversity in NL, less measurements, or even strain-level variation. Indeed, we observed two immunomodulatory taxa with significant strain divergence from NL, showing moderate (*Akkermansia*, Suppl. Fig. 7a, c, *P* = 0.07) to strong phylogenetic associations with cytokine responses (*Streptococcus salivarius*, Supplementary Fig. 7b, d, *P* < 10^−6^).

Overall, intracellular pathogens and cytokines produced by myeloid cells tended to have a larger number of microbiome associations, while lymphocyte-derived cytokines showed greater microbiome dependence in combination with extracellular stimuli (Fig. 3e, Supplementary Data 10). TNF-α showed the strongest microbiome dependence when stimulated with *C. albicans*. *A. muciniphila*, and *B. longum* had the strongest positive effects, while rural hallmark *P. copri* showed a reverse pattern. *B. longum* and *P. copri* showed respective positive and negative regulation of TNF-α, but an inverse pattern in IL-1β when stimulated by *E. coli*, *C. burnetii*, or *S. typhi*. Despite their low prevalence, Ascomycota were associated with decreased response to TNF-α response to *C. albicans*, suggesting potential intra-kingdom tolerance (Supplementary Fig. 8). Thus, differences in intestinal microbiota driven by residency and its ramifications^7^ contribute to variability in immune responses.

### Pathways with microbial enzymes differentiate the urban and rural metabolome and associate with cytokine responses

**Fig. 4:**
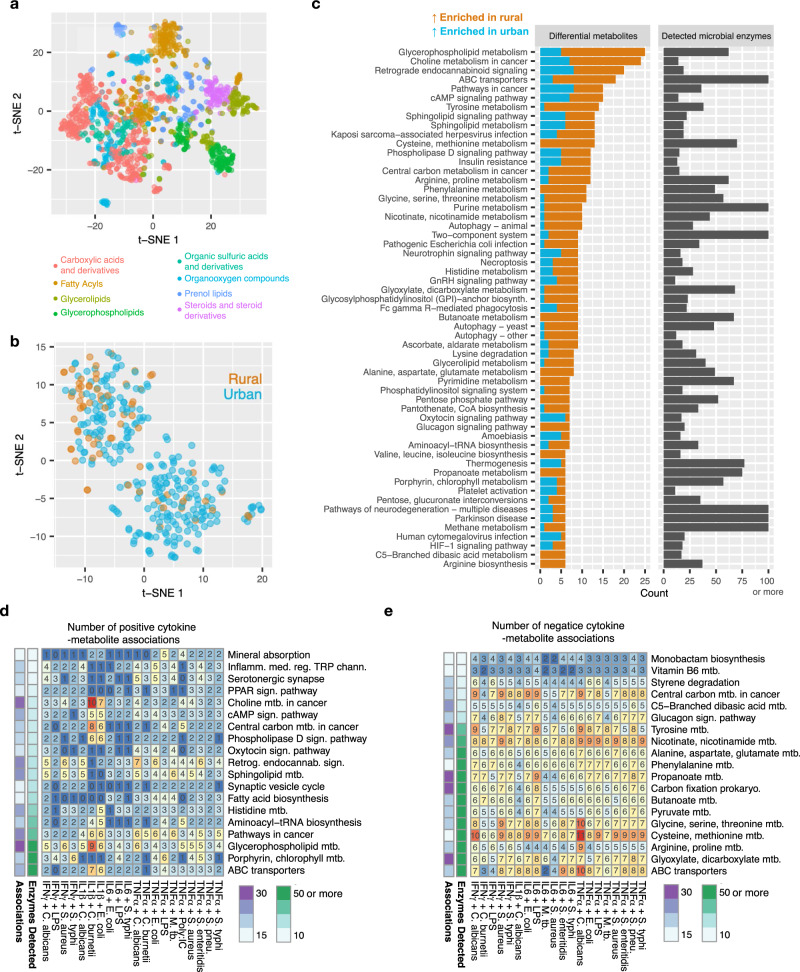
Pathways with microbial enzymes differentiate the urban/rural metabolome and associate with cytokine responses. **a** The intensities of 1607 metabolites were standardized (z-scored) across the samples and projected with t-SNE. K-means cluster was used to discover 28 metabolite clusters (“Methods”) and annotated with the most prevalent HMDB molecular class. **b** t-SNE plot of samples metabolome profiles separating urban and rural samples. **c** Differentially abundant metabolites between urban and rural samples (Wilcoxon rank-sum two-sided test, *P*_Bonferroni_ < 0.05), stratified by KEGG pathways. **d**, **e** Number of positive and negative effects metabolites on cytokine expression, counted by detected KEGG pathways. Pathways with at least 10 different microbial enzymes in the entire cohort are selected. Metabolite-cytokine correlations are determined by Spearman correlation test subject to *P*_FDR_ < 0.05. Source data are provided in the Source data file.

Urbanization-driven differences in immune responses and the impact of immunomodulatory species were further investigated through circulating metabolites. Untargeted plasma metabolome profiling using liquid chromatography–mass spectrometry (LC–MS) identified 1607 mass/charge (*m*/*z*) peaks corresponding to known molecules (Supplementary Fig. 9a). We used database integration to alleviate the uncertainty in compound identification, as correlated abundances of multiple ions can reflect coherent metabolic pathway activities^34^. First, co-abundance clustering was performed as a quality control and revealed 28 metabolite clusters, dominated by fatty acyls, carboxylic acids, and derivatives, as well as prenol lipids and glycerophospholipids (Fig. 4a, Supplementary Fig. 9b, Supplementary Data 11; metabolite classes as in HMDB). Second, a total of 632 peaks were matched to one or more KEGG compound identifiers, which were linked to 351 KEGG pathway identifiers (Supplementary Data 12). Finally, to approximate microbial influences on circulating metabolites, we utilized the stool metagenomes and KEGG Orthogroup annotation to count the number of detected microbial enzymes for each of the 351 pathways. This yielded 325 pathways with at least one microbial enzyme.

The metabolome profiles differentiated the urban and rural samples (Fig. 4b, Supplementary Data 11), particularly in the secondary metabolites, as also recently reported by our group^33^. Surprisingly, rural samples showed a larger number of increased metabolite intensities. Pathways with more than ten microbial enzymes that included differential metabolites included glycerophospholipids, sphingolipid and cholesterol products, and amino acids including histidine, tyrosine, methionine, and cysteine (Fig. 4c, Supplementary Data 11). Rural samples had a higher intensity of potentially dietary-related compounds, including ascorbic acid (vitamin C), fumaric acid, citric acid, L-Aspartic acid, as well as formiminoglutamic acid, an intermediate in histidine catabolism.

Correlation analysis revealed 1234 metabolites associated with cytokine expression (*P*_FDR_ < 0.05, Spearman correlation, Supplementary Fig. 9c, Supplementary Data 13). TNF-α stimulated by *C. albicans* was associated with most metabolites (685), concordant with the microbiome analysis above. Positive immunomodulatory effects were mediated by microbially-modified metabolites including histidine, as well as glycerophospholipids and sphingolipids (Fig. 4d, Supplementary Data 13). Negative effects were predominantly manifested through products of tyrosine, alanine, phenylalanine, and cysteine metabolism (Fig. 4e, Supplementary Data 13).

Numerous individual molecules associated with cytokine responses have known immunomodulatory roles (Supplementary Data 13). For example, itaconate is a host macrophage-produced inhibitor of inflammation^35^ that activates Nrf2 via KEAP1. The former act in a nitric oxide-producing pathway in response to pathogens. Low concentrations of itaconate, which can be derived by fungal fermentation^36^, were associated with increased TNF-α response to *S. enteritidis*, *E. coli*, LPS, *S. typhi*, *S. pneumoniae*, and *M. tuberculosis*, as well as IFN-γ responses to *C. burnetii* and *S. typhi*, reflecting the expected role of this metabolite in downregulation of inflammation. Glyphosate, an environmental toxin^37^, was also associated with lower TNF-α stimulation by *S. aureus* and *E. coli*.

### Correspondence between metabolomics and immunomodulatory species’ enzymes reveals affected pathways

**Fig. 5:**
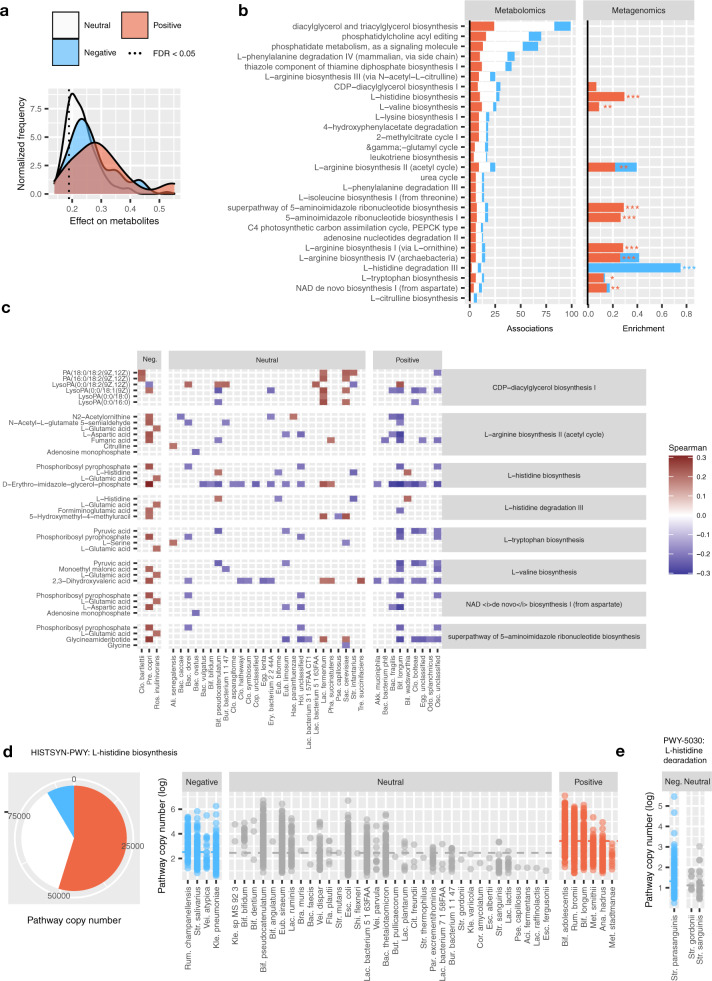
Integrative analysis of metabolomics and metagenomics uncovers histidine and arginine metabolism pathways. **a** Distribution of per-species effects on the plasma metabolome. Species are stratified by immunomodulatory effects derived from the log-linear model described above. For each species, the maximum absolute Spearman coefficient with 1607 measured metabolites is chosen and the significance threshold is determined subject to *P*_FDR_ < 0.05. **b** Metabolites affected by immunomodulatory species, organized by MetaCyc pathways. The bars show the number of significant correlations per species type (left, Spearman correlation test, *P*_FDR_ < 0.05) and significant effects from pathway copy number model (right, *P < 0.1, **P < 0.01, ***P < 0.001). Species colors as in (**a**). **c** Pathways with significantly enriched copy numbers in positive or negative species and significant correlations with plasma metabolites (Spearman correlation, *P*_FDR_ < 0.05). **d** Copy numbers of histidine biosynthesis pathway (MetaCyc, HISTSYN-PWY) and **e** histidine degradation III pathway (PWY-5030) detected in the metagenome of negative (blue), neutral (gray), and positive (red) species. Source data are provided in the Source data file.

An indirect validation of immunomodulatory species is performed by gauging their effects on independently-measured circulating metabolites. We approximate this effect by computing the maximum Spearman correlation between each microbes’ abundance and any one metabolite intensity. Indeed, the distribution of the approximated effects proved significantly greater for significant species (Fig. 5a, Supplementary Data 14), particularly the 21 positive species. This suggests that the 34 species affecting cytokine expression also have a greater imprint on the plasma metabolome, which is consistent with microbiota-affected immune response models^38^. In total, 28 MetaCyc pathways entailed at least three metabolites with significant associations with immunomodulatory species (Fig. 5b, left; Supplementary Data 13), suggesting possible mechanisms manifested in multiple interdependent compounds.

We then asked which of the identified 28 pathways might be significantly encoded in the genomes of immunomodulatory species (Fig. 5b, right; Supplementary Data 15). We devise a model of pathway copy number enrichment in positive or negative species compared to neutral (“Methods”). Ten of the metabolite-associated pathways were enriched in immunomodulatory species’ genomes, where the majority, nine, were encoded by the positive species. The resulting nine pathways include microbially-modified amino acids, for example arginine and histidine, which were also seen in associations with urbanization and cytokines, as described above.

A detailed look at compounds from the pathways reveals the following general pattern: negative correlation between positive species and compounds, positive correlation for negative species, and mixed effects for neutral species (Fig. 5c, Supplementary Data 14). The largest number of associations, 15, was contributed by *Bifidobacterium longum*, the microbe with strongest positive immunomodulatory effects as seen above. In histidine metabolism, *B. longum* showed a negative association with Phosphoribosyl pyrophosphate and D-Erythro-imidazole-glycerol-phosphate, the latter showing a strong dependence along the negative-neutral-positive species spectrum.

More than half of the histidine biosynthesis copies were contributed by positive species, including *B. longum* and *R. bromii* (*P* < 1.37E−10, Fig. 5d). Conversely, negative immunomodulatory species were enriched for histidine degradation (*P* < 5.6E−10, Fig. 5e), the latter encoded almost exclusively by *Streptococcus parasanguinis*. The associations between immunomodulatory species and metabolites can thus be traced to metagenomic pathways, whereas the differential compound intensity may result from a combination of strain variation (*R. bromii, Akkermansia*) or relative abundance alone (*B. longum*).

### Integrative analysis details microbial mechanisms in histidine and arginine metabolism pathways

**Fig. 6:**
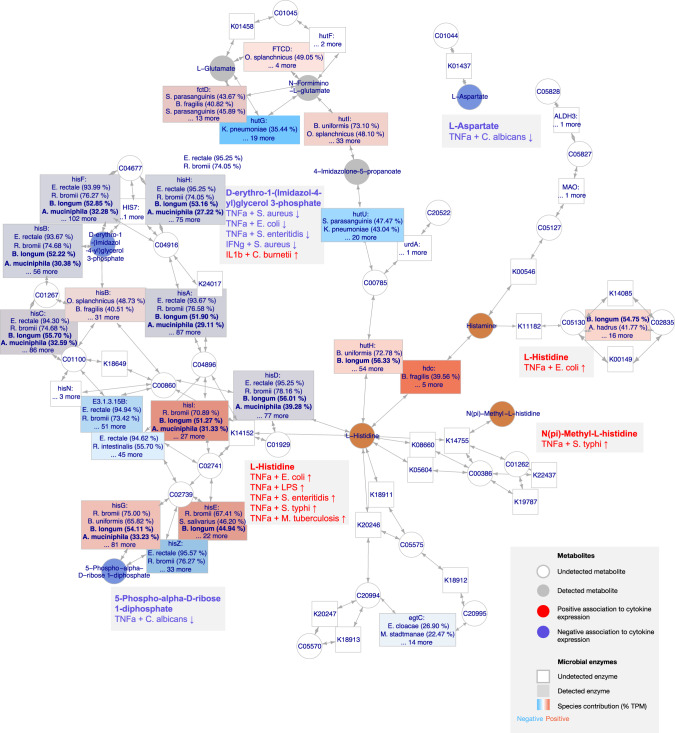
Detected enzymes, metabolites, and associations in the histidine metabolism pathway (KEGG KO00340). Colored nodes represent detected microbial enzymes (squares) and compounds (circles), with significant correlations with cytokine responses in red (positive) and blue (negative). Enzymes detected in stool microbiomes are shown as colored squares, with contribution from negative (blue) or positive species (red) quantified as a fraction of total RPKM in the cohort per species type. Percentages in squares show prevalence of enzyme encoded by each species. Source data are provided in the Source data file.

We outline histidine metabolism as a predicted microbial amino acid metabolism pathway affecting cytokine responses by combining multiple lines of evidence above. Metagenomics and metabolomics measurements detected 26 enzymes and 9 circulating compounds corresponding to KEGG pathway histidine metabolism (KO00340) (Fig. 6). Histamine, histidine, and N(pi)-Methyl-L-histidine had positive effects on TNF-α stimulation with all Gram-positive bacteria, while L-Aspartate, D-Erythro-imidazole-glycerol-phosphate, and 5-Phospho-alpha-D-ribose 1-diphosphate had the corresponding negative effects.

Enzymes directly involved with histidine were predominantly encoded by positive immunomodulatory species (*hisD*, *R. bromii*, *B. longum*; *hutH*, *B. longum*; *hutC*, *B. uniformis,* and *B. longum*). Compounds resulting from histidine catabolism, D-Erythro-imidazole-glycerol-phosphate and 5-Phospho-alpha-D-ribose 1-diphosphate are directly involved with gene cluster *hisA-hisH* encoded both negative immunomodulatory species (*E. rectale*, 93.7–95.3% samples), but also *B. longum* (44.9–56.0%) and *A. muciniphila* (27.29–32.2%). Since *P. copri* showed a strong reverse positive effect on Phosphoribosyl pyrophosphate and D-Erythro-imidazole-glycerol-phosphate (Fig. 5c, Supplementary Data 14), but had no enzymes in the histidine metabolism pathway, this association may stem from reverse, mutually exclusive abundance patterns with positive immunomodulatory species.

A pathway that downregulates cytokine production that emerged from metabolomics and metagenomics was arginine biosynthesis (KEGG pathway KO00220). Three of its compounds negatively-associated with relative abundance of *B. longum* and TNF-α response to *C. albicans*: L-Aspartate, fumarate, N-Acetylornithine. *B. longum* was also strongly enriched in pathway copy numbers (*P* < 0.002, Supplementary Fig. 10a-b), encoding genes *argB*, *argC*, *argD*, *argG*, *argH*, and *argJ* in the *arg* family, previously observed in limiting T-cell functions^39^. While *B. longum* contributed arg genes in 53.8–55.6% samples, *A. muciniphila* also encoded *argB, argC, argD,* and *argH* in 32.9–35.8% of samples (Supplementary Fig. 10c). Genes *argD, argG,* and *argH* are directly involved in reactions with N-Acetylornithine, L-Aspartate, and fumarate, respectively. While limited enzymes and compound detection may not allow for narrowing down of immunomodulatory factors to a single microbial molecule, the evidence from cytokine expression, circulating metabolites, and metagenomic pathway copy numbers suggest an active role of *B. longum* in histidine and arginine metabolism.

## Discussion

Repercussions of urbanization, including changes in diet, increased hygiene, and antibiotic usage, can have positive short-term, but unknown long-term effects on health. Traditional, rural populations can be seen as examples of past habits regarding diet, medication, and environmental exposure. Our gradient analysis shows a progression in intestinal microbial compositions and exposing prominent shifts in a number of taxa. Rural samples show a steady increase in *Prevotella copri*, which is implicated in decreased cardiometabolic disease risk and insulin resistance^32,40^. Conversely, while the abundance of *A. muciniphila* is linked to obesity-risk genes variants and protective obesity effects^41,42^, Verrucomicrobia are rarely found in African traditional populations^1^. Since Western fiber-poor diets promote mucin degraders^43^ and a thinner mucus layer is characteristic in inflammatory environments^44^, the presence of *A. muciniphila* is consistent with increased pro-inflammatory cytokine expression in urban individuals (Supplementary Fig. 5). Even though the rural lifestyle can be perceived as healthier in general, the beneficial effects and survival of individual microbes are context-specific and require targeted experiments.

Urban samples were more similar to Dutch samples, which might reflect a transition to Western-type diet, different hygiene, and microbe exposure^22^. The gradient was also noted in an independent strain-level analysis, uncovering 19 diverging genomes that may bring additional gene- or SNP-level phenotypes beyond the scope of this study. Interestingly, rural samples harbored a notable proportion of fungi. Since fungi were significantly depleted in urban samples and barely detected in the Dutch cohort (analyzed with the same metagenomics protocols), it suggests the potential increased susceptibility to fungal colonization or favorable growth conditions in Tanzanian intestinal ecosystems. Recent parasitological data has also revealed that gut eukaryotes impact bacterial diversity in West African rural populations^22^, uncovering another previously unexplored microbial influence.

Given all of the above, it is unsurprising that urban and rural populations have contrasting metabolomic and immune profiles. This may be in part, but not entirely, attributed to genetics^45^. As we have recently reported that these rural and urban populations from Tanzania do not represent two distinct genetic clusters^33^, most differences likely stem from the environment. The most strongly influenced cytokines were TNF-α, IFN-γ, and IL-1β, while IL-6 and IL-10 were affected to a lesser degree. The most affected stimulation by the urban-rural axis, metabolites, and immunomodulatory species was TNF-α with *C. albicans*, which also had the highest explained variance from the microbiome in the Dutch cohort^46^. Recently, it was shown that *Bacteroides thetaiotaomicron* and *Lactobacillus reuteri* shape the mucosal localization of *C. albicans*^47^. Hence, the variation in immune response to this commensal fungus may stem from direct physical interaction in the mucus layer. Pathway analysis of this same cytokine response also uncovered *S. cerevisiae* enzymes in sphingolipid metabolism, opening the possibility of interesting inter-kingdom effects.

A wide panel of stimuli and a number of measured cytokines allows us to assess a range of immune system modalities, as well as increase the statistical power. Several microbes have previously been reported as modulating the immune system, the metabolome or both. Positive immunomodulators *B. longum* and *A. muciniphila* produce inosine and their colonization affects outcomes in cancer immunotherapy^48,49^. Isolates from the *Eggerthella* genus were recently shown to promote accumulation of Th17 cells in a highly strain-specific manner^50,51^. Increases in *R. bromii, E. rectale,* and *Roseburia* occur with a plant-based diet in humans. Expansion of *Bilophila*, which has been linked to an increase in Th1 cytokines and susceptibility to colitis in mice^52^, is linked to high-fat animal diet^53,54^. In addition, some of the species we identified have been linked to inflammatory intestinal disease. These include *Prevotella copri*, which shows an increased and steady abundance in Crohn’s disease (CD), and *Klebsiella pneumoniae* which plays a role both in dysbiotic ulcerative colitis and CD^19^. While strongly significant, a number of Firmicutes might also reflect the general propensity of the urban samples to express higher levels of cytokines.

Circulating metabolites had strong associations with cytokine responses, and were more affected by independently identified immunomodulatory species, confirming the intricate connection between microbial metabolism in the gut and the immune system. It is important to note that the high correspondence between molecules of the same class may reflect both genuine metabolic flow and a potential bias in detection of molecules with similar physico-chemical properties. We used pathway enrichment considering all possible identities for a compound, which can alleviate some of the errors coming from identification uncertainty if each compound were considered in isolation^55^. Also, any molecule on a pathway might contribute to the phenotype, as do many currently unknown intermediates^56–58^. In addition, our study sampled microbes residing in the gut, while metabolites were measured directly in the circulation. The effects of microbial abundance on plasma metabolite levels are limited and hold only for certain metabolite classes^59^, which was evident in our joint metagenomic/metabolomic analysis that was skewed toward essential amino acids and their derivatives. Associations between microbes and metabolites can arise from indirect modification, for example in the case sphingolipids, which showed strong immunomodulatory effects, and are produced by host and gut eukaryotes^60,61^, but modified by *Bacteroides*^56,57^.

The listed limitations require careful treatment of individual associations and combining multiple measurements in order to identify causal mechanisms. Differential metabolites between urban and rural samples, associations with cytokine expression and immunomodulatory species abundance, and metagenomic pathway enrichment, all point to histidine metabolism. The published analysis on the 500 FG cohort showed congruent results in several amino acids, including inosine, methionine and also histidine^17^. In particular, histidine can be decarboxylated in both mammalian and gut bacterial cells to form histamine, which engages with receptors H1R-H4R and supports Th1 and Th2 cell polarization^62^. We detected both histamine and histidine as having positive associations with cytokine expression as well as being encoded by positive immunomodulatory species. An additional mechanism supported by our data was arginine metabolism, where *B. longum* and *A. muciniphila* encoded *arg*, a conserved group of enzymes that modify the host arginine as a survival mechanism^63^. This gene family is associated with regulation of host T-cell functions by myeloid suppressor cells^39^. Our data offer a complementary, functional view and confirms that histidine and arginine metabolism are influenced by microbes in vivo and might in turn affect circulation and immune responses.

Multi-omics data derived from diverse cohorts increase statistical power to detect causal immunomodulatory mechanisms. Concurrently, improved computational tools are required to explore the vast space of unknown metabolites, where small molecular changes can have huge downstream consequences. Taken together, mechanistic understanding of microbial metabolism and its downstream consequences on the immune system can uncover new personalized or population-specific therapeutic interventions.

## Methods

### Sample handling and sequencing

Sample processing and whole blood stimulations and whole blood counts were performed at Kilimanjaro clinical research institute (KCRI), Moshi, Tanzania. Plasma samples for metabolomics, supernatants of the whole blood stimulations, and stool for metagenomics were transported to Radboud University Medical Center (Radboudumc) on dry ice.

Stool samples were stored at −80 °C prior to nucleic acid extraction using the AllPrep 96 PowerFecal DNA/RNA kit from QIAGEN (custom product # 1114341). This method pairs bead-beating on a Tissuelyser II (QIAGEN) with a 96-well AllPrep protocol and is available through QIAGEN. Bead-beating is performed twice at 20 Hz for 5 min each round with a rotation of the plate in between rounds. Purified DNA was stored at −20 °C. Metagenomic sequencing libraries were prepared from 2 ng of input DNA using the Nextera XT DNA Library Preparation kit (Illumina) according to the manufacturer’s recommended protocol. Prior to sequencing, libraries were pooled by collecting equal volumes of each library. Insert sizes and concentrations for each pooled library were determined using an Agilent Bioanalyzer DNA 1000 kit (Agilent Technologies) prior to sequencing on an Illumina NovaSeq 6000 with 151 bp paired-end reads to yield ~10 million paired-end reads per sample. Data were analyzed using the Broad Picard Pipeline which includes de-multiplexing and data aggregation (https://broadinstitute.github.io/picard).

### Metagenomic data processing

Samples that were included in the metagenomic analyses were required to have at least 4 million sequencing reads, resulting in 315 samples (*n* = 315). Reads were first processed using KneadData (http://huttenhower.sph.harvard.edu/kneaddata). This included quality-trimming (trimmomatic parameters: MAXINFO:90:0.5), read-filtering based on a minimum read length of 60 bp, and removal of potential human contamination by filtering reads that aligned to the human genome (reference genome hg19). Quality-controlled, paired-end reads were aligned against a database of unique clade-specific marker genes using Bowtie2 and taxonomic profiles were inferred with MetaPhlAn 2.2^64^. For subsequent analysis, we used species, genus, and phylum compositions, represented as relative abundances of taxa within each sample.

Functional profiling was performed using HUMAnN2 (http://huttenhower.sph.harvard.edu/humann2). Briefly, reads are mapped against a customized database of functionally annotated pangenomes, only considering organisms that were identified during the taxonomic profiling step. Functional annotation of the protein sequences in the pangenomes to their respective UniRef90 family is provided with the software. Reads that cannot be mapped are subsequently aligned against the complete UniRef90 database. The proteins are further mapped to KEGG Orthogroups (KOs). The community totals are computed for each protein family (RPK) and converted into relative abundances. For subsequent downstream analysis, these tens of thousands of gene families were further grouped into broader functional categories: MetaCyc metabolic pathways. This process yielded 212 species present in at least 10 samples, 82 genera, 7658 KOs, and 486 MetaCyc pathways.

### Metagenomics statistical analyses

#### Differential abundance of taxa

The factors that affected microbiome compositions as a whole were identified with PERMANOVA (R package vegan, function adonis, significant level *P* < 0.001). Differential abundance of phyla, species, and metagenomic pathways between Tanzanian and Netherlands cohorts, as well as urban and rural samples were computed with R package Maaslin 2, version 1.0.0 (https://huttenhower.sph.harvard.edu/maaslin/). The recommended multiple testing-adjusted significance value of *P*_FDR_ < 0.25 was applied. We considered only the species detected in both cohorts in the following differential abundance tests. The species changing according to the rural-urban-Netherlands gradient were determined by using Wilcoxon rank-sum test twice. Significance was determined as larger of the *P*-values when comparing species relative abundance difference between (1) rural versus urban and (2) urban versus Netherlands samples. The species were further filtered subject to prevalence >20% and *P*_FDR_ < 0.05. The two alternative orderings (urban-rural-Netherlands and rural-Netherlands-urban) were tested in the same manner.

#### Dimensionality reductions

All ordination plots were derived using Jensen-Shannon divergence (R package philentropy, version 0.4) on relative abundance of 212 profiles (TZ samples only) or 178 shared species (TZ and NL samples combined). The distance matrix was projected to two dimensions using Multidimensional scaling (TZ) or t-SNE (TZ and NL). Number of clusters was determined based on the Jensen-Shannon divergence and a consensus hierarchical clustering with R package pvclust, version, using 100 bootstrap runs. The number of clusters was determined when two subsequent height cutoffs differed by less than twofold, yielding three clusters C1, C2, and C3.

#### Strain divergence analysis

Strain divergence analysis. Strain-level profiling was obtained by collecting raw metagenomic reads from both TZ and NL cohorts (796 samples in total) and running Strainphlan^65^ with default parameters. A total of 68 taxa had sufficient read coverage to derive phylogenetic trees. For each taxon, its strain divergence was estimated by explained variance of residency (rural, urban, NL) in underlying phylogenetic distance matrix using PERMANOVA^66^.

#### Associations between microbiome and metadata

Cluster assignment was compared to categorical variables using the Chi-square test and threshold *P* < 0.05. Age was converted to three equal-frequency bins. Significant changes in phylum abundance were estimated using the R package Maaslin 2 (version 1.0), treating each variable as a fixed effect and applying the recommended threshold *P*_FDR_ < 0.25.

Dependence of 17 dietary variables was compared with the microbiome clusters C1–C3, residency, presence of fungi. Chi-square test (*P* < 0.05) was used for categorical variables (alcohol, milk, meat, and consumed intestine type) and Kruskal–Wallis (*P* < 0.05) for variables quantifying weekly consumption of various types of food (meat, ugali, rice, banana, wheat, potato chips, fish, beans, vegetables, fruits) and drink (carbonated soda, sweet tea, milk).

#### Linear models of cytokine responses

Immunomodulatory species were selected by using the measured expression of five cytokines (*c;* IL-6, TNF-α, IFN-γ, IL-1β, and IL-10), stimulated with nine stimuli (*s*; LPS, *S. aureus*, *S. pneumoniae*, *S. typhi*, *S. enteritidis*, *C. burnetii*, *E. coli*, *M. tuberculosis*, and *C. albicans;* Poly:IC excluded), and stool samples of subjects (*i* = 1, 2,…, 299; subjects with at least one cytokine response and microbial profile measured). The data consist of 12,348 observations (out of possible 5 × 8 × 299 = 13,455 due to missing measurements). The cytokine expressions *y*_c,s,i_ were fit using the log-linear model:

1 $$\log \,{{{{{{\rm{y}}}}}}}_{{{{{{\rm{c}}}}}},{{{{{\rm{s}}}}}},{{{{{\rm{i}}}}}}}={{{{{{\rm{\beta }}}}}}}_{0}+{{{{{{\rm{\beta }}}}}}}_{{{{{{\rm{c}}}}}},{{{{{\rm{s}}}}}}}+{{{{{{\rm{\beta }}}}}}}_{{{{{{\rm{a}}}}}}}{{{{{{\rm{age}}}}}}}_{{{{{{\rm{i}}}}}}}+{{{{{{\rm{\beta }}}}}}}_{{{{{{\rm{s}}}}}}}{{{{{{\rm{sex}}}}}}}_{{{{{{\rm{i}}}}}}}+{{{{{{\rm{\beta }}}}}}}_{{{{{{\rm{m}}}}}}}{{{{{{\rm{mic}}}}}}}_{{{{{{\rm{m}}}}}},{{{{{\rm{i}}}}}}}+{{{{{{\rm{\varepsilon }}}}}}}_{{{{{{\rm{c}}}}}},{{{{{\rm{s}}}}}},{{{{{\rm{i}}}}}}}.$$

with the following parameters: *ε*_c,s,i_ is the unexplained variance, *β*_0_ the intercept, *β*_c,s_ the interaction between cytokine *c* and stimulus *s*, and *β*_a_, *β*_s_, and *β*_m_ coefficients for age, sex, and microbe abundance, respectively. The subject-specific variables are age_i_ (continuous), sex_i_ (discrete), and mic_m,i_ the relative abundance of the microbe *m*, converted to ranks (where no abundance amounts to rank 0 and maximum abundance to rank 298). Despite the observations not being entirely independent (each subject can be repeated in multiple cytokines and stimuli) we chose to treat them as such rather than average across cytokines and stimuli, treating them as separate experiments that bear additional information about the immune response. We alleviate the effect of potential sample-specific (latent) factors by using a stringent multiple testing threshold stated below. A comparison with linear mixed effect models yielded consistent results (see the code repository).

To account for co-abundance effects, the model was fit once for each microbe, using the R function lm. We tested 111 microbial species with at least 20% prevalence (*m* = 1, 2,…,111). The species were deemed immunomodulatory if *β*_m_ had a significant contribution after stringent multiple testing adjustment (*P*_Bonferroni_ < 0.005, T-test). Based on significance and sign of corresponding *β*_m_, species were categorized as positive (implying an increase in overall cytokine response), negative (decreased cytokine response), or neutral (non-significant).

The same approach was used to test for immunomodulatory species in the 500 FG cohort, which had 473 samples and macrophage expressions of IL-6 and TNF-α, stimulated with LPS, *C. albicans*, *M. tuberculosis*, and *S. typhi*, totaling 3766 observations. We tested 176 species detected in both Tanzania and 500 FG cohorts.

#### Linear model of metabolic pathway copy number contribution

Metabolic pathway modules encoded by microbial species were quantified by Humann2^67^ and encoded as MetaCyc identifiers, yielding 486 pathways (*p* = 1, 2,…, 486), contributed by 212 different microbes (*m* = 1, 2,…, 212) in 315 samples with detected pathways (*i* = 1, 2,…, 315). Microbial species were further categorized on their immunomodulatory effect (*e*) as positive (21 species), negative (13), and neutral (178), as described above.

Pathway abundance in each subject is quantified as the number of complete “copies” of the pathway encoded by each species, normalized by pathway length as described previously^67^, and represented here as *y*_p,m,i_. The variation in *y*_p,m,i_ was fit using the log-linear model

2 $$\log \,{{{{{{\rm{y}}}}}}}_{{{{{{\rm{p}}}}}},{{{{{\rm{m}}}}}},{{{{{\rm{i}}}}}}}={{{{{{\rm{\beta }}}}}}}_{0}+{{{{{{\rm{\beta }}}}}}}_{{{{{{\rm{d}}}}}}}+{{{{{{\rm{dep}}}}}}}_{{{{{{\rm{i}}}}}}}+{{{{{{\rm{\beta }}}}}}}_{{{{{{\rm{m}}}}}}}{{{{{{\rm{mic}}}}}}}_{{{{{{\rm{m}}}}}},{{{{{\rm{i}}}}}}}+{{{{{{\rm{\beta }}}}}}}_{{{{{{\rm{e}}}}}}}\,{{{{{{\rm{eff}}}}}}}_{{{{{{\rm{m}}}}}}}+{{{{{{\rm{\varepsilon }}}}}}}_{{{{{{\rm{p}}}}}},{{{{{\rm{m}}}}}},{{{{{\rm{i}}}}}}}.$$

with the following parameters: *ε*_c,s,i_ is the unexplained variance, *β*_0_ the intercept, *β*_d_ the subject sequencing depth, *β*_m_ microbe abundance, and *β*_e_ the microbes’ immunomodulatory effect. The subject-specific variables are represented as dep_i_, the logarithm of total number of reads detected in subject *i*, and mic_m,i_ the relative abundance of the microbe *m* in subject *i*. The immunomodulatory effect eff_m_, is a categorical variable associated with each microbe *m*, where neutral is taken as the reference value (absorbed by the intercept).

The model is fit independently for each pathway *p*. We use the T-test to quantify the significance of the *β*_e_ coefficients. These reveal an over- or under-representation of gene copies mapping to pathway *p* in positive or negative species.

### Untargeted plasma metabolomics

Plasma for metabolomics was transported to General Metabolics, LLC (Boston, USA) on dry ice and measured as previously described^68^. Briefly, untargeted liquid chromatography–mass spectrometry (LC–MS) was used to measure metabolites from plasma samples using a high throughput flow-injection mass spectrometry technique. This platform consists of an Agilent Series 1100 LC pump coupled to a Gerstel MPS2 autosampler and an Agilent 6550 Series Quadrupole Time-of-flight mass spectrometer (Agilent, Santa Clara, USA) equipped with an electrospray source operated in negative and positive mode. With this platform 50–1000, ion *m*/*z* (mass‐to‐charge ratio) can be detected with a flow rate of 150 μL/min and 1.4 Hz in 1 min cycle time.

Data were further analyzed by performing centroiding on a high-performance computing cluster using the bioinformatics function in Matlab R2018a (The Mathworks, Natick). Centroiding was done once for each sample on the total profile spectrum obtained by summing all single scans recorded over time and using wavelet decomposition. A cut-off of 500 ion counts was applied to avoid detection of too sparse features. Centroid lists from different samples were merged to a single matrix by binning the accurate centroid masses within the tolerance given by the instrument resolution (0.001 amu). The functionality of this proprietary workflow is described in more detail in ref. ^68^.

A list of putative metabolites was annotated with a series of analysis strategies including deisotoping, decluttering, adduct detection, and library matching in KEGG, HMDB, and CHEBI databases. The monoisotopic mass for the neutral molecule was calculated, and common ESI ions/adducts for the monoisotopic masses and isotopes were generated. For each measured ion, all possible hits among theoretical ion masses within a tolerance of 0.003 amu were considered. This resulted in a data matrix of 323 individual samples and 1607 compounds/metabolites (peaks mz/rt).

#### KEGG pathway annotation

A total of 1203 compounds were linked to a KEGG compound. identifiers, from which of 680 compounds were linked to 351 KEGG pathways (KOs), containing 10,060 enzymes (KOs).

#### MetaCyc pathway module annotation

The MetaCyc database downloaded as of Oct 13, 2020. A total of 336 metabolites, represented as KEGG compound IDs, were found to participate in 7545 cataloged reactions and 2074 pathway modules.

### Metabolomics statistical analysis

#### Quantification, quality control, and clustering

Metabolite intensity was standardized (z-scored) across samples. Clusters were obtained using k-means. The number of clusters was determined by optimal matching to the molecular classes (from HMDB database) using adjusted random index (ARI) measure. Low-dimensional projection was obtained using t-SNE^69^.

#### Correlation analysis

Metabolite intensities were compared to relative species abundance and cytokine expressions using Spearman correlation subject to *P*_FDR_ < 0.05.

### Whole blood stimulation assays and cytokine quantification by ELISA

Blood sample collection, processing, and ex vivo cytokine production experiments were performed at the biotechnology laboratory facility available at Kilimanjaro clinical research institute (KCRI) in Moshi, Tanzania. A whole blood count with leukocyte differentiation was measured on a Sysmex XN-450 Hematology Analyzer (Sysmex Corporation, Kobe, Japan). Whole blood was stimulated with bacterial and fungal stimuli and TLR3 and TLR4 agonists. The stimulation experiments were performed as follows: 100 μl of heparin blood was added to a 48-wells culture plate and subsequently stimulated with 400 μl of stimulus for 48 h at 37 °C and 5% CO_2_.

The bacterial and fungal stimuli were cultured and frozen at Radboudumc and then shipped to KCRI. Their respective concentrations were as follows: PHA (10 μg/ml, Sigma), LPS (100 ng/ml, Sigma), Poly:IC (50 μg/ml, Sas Invivogen), *Mycobacterium tuberculosis* (5 μg/ml, H37Rv, in-house), *Coxiella burnetii* (10^7^ CFU/ml, Nine miles/RSA493), *Escherichia coli* (10^6^ CFU/ml, ATCC35218, in-house), *Staphylococcus aureus* (10^6^ CFU/ml, ATCC29213, in-house), *Candida albicans* (10^6^ CFU/ml, UC820, in-house), *Streptococcus pneumonia* (10^7^ CFU/ml, TIGR4, in-house), *Salmonella typhimurium* (10^6^ CFU/ml, Phage type 510, in-house), and *Salmonella enteritidis* (10^6^ CFU/ml, in-house). Stimuli were prepared in RPMI culture medium (Dutch modified, Invitrogen) supplemented with 50 µg/mL gentamicin, 2 mM Glutamax, and 1 mM pyruvate.

Supernatants were collected and stored at −80 °C until used for ELISA. The concentrations of cytokines were quantified in the supernatants using ELISA according to the instructions (given IL-6, IL-1β, IL-10, and tumor necrosis factor (TNF-α): R&D Systems; interferon (IFN-γ): Sanguin). All samples were measured using kits of the same lot number.

### Description of the study area and population

This study is part of the Human Functional Genomics Project (www.humanfunctionalgenomics.org). The study protocol was approved by the Ethical Committees of the Kilimanjaro Christian Medical University College (CREC) (No. 2443) and the National Institute for Medical Research in Tanzania (NIMR/HQ/R.8a/Vol. IX/2290 and tNIMR/HQ/R.8a/Vol.IX/3318). In the Netherlands, the study was approved by the Ethical Committee of the Radboud University Medical Centre Nijmegen (CMO Arnhem-Nijmegen; 2016-2657). A total of 323 Tanzanian healthy individuals aged between 18 and 65 years living in the Kilimanjaro region in Northern Tanzania were enrolled between March and December 2017. Participants enrolled at the Kilimanjaro Christian Medical Center and Lucy Lameck Research Center, in Moshi municipal, a capital and commercial city of the Kilimanjaro region with over 200,000 inhabitants. Moshi city comprises diverse ethnic groups, economic status, and lifestyle, whereby most inhabitants have adopted a Western lifestyle. People living in the Moshi town (urban area) have access to good sanitation with universal coverage of chlorinated tap water and toilet facilities. In contrast, people we designated as living in a rural area represent the rural population of Tanzania; people live within large family units, whereby the economy depends mainly on subsistence farming and animal husbandry. Most individuals in the rural area belong to the Chagga tribe and follow a traditional lifestyle whereby using pit latrine and consumption of starch and vegetable diet are common. They also have access to good sanitation and water primarily from the slope of Mount Kilimanjaro or from wells. In this study, people from both urban and rural areas were enrolled. Since the rural individuals all belong to the Chagga tribe, a higher number of urban participants were enrolled to alleviate confounding from a potentially different genetic composition between the two sub-cohorts.

The study information was given through leaflets or announced during the mass gathering. Volunteers were pre-screened by a member of the study team before being invited to the study center. Exclusion criteria were pregnancy, a known acute or chronic disease, use of antibiotics or antimalarials in the previous 3 months, or receiving treatment for tuberculosis infection in the past year. The eligible individuals who gave written informed consent to participate in the study were screened for HIV infection (SD BIOLINE HIV-1/2 3.0 kit, Standard Diagnostics, Kenya, #03FK16) and malaria (Malaria Pf/PAN (HRP2/pLDH) Ag Combo RDT kit, Accessbio, USA, #RMRM-02571) and random blood glucose (ACCU-CHECK glucose test strips, Roche Diabetes Care GmbH, Germany, #07124112036), blood pressure, body weight, and height were measured. We excluded individuals with a positive HIV or malaria rapid test, a blood pressure ≤90/60 mmHg or ≥140/90 mmHg, or with random blood glucose >8.0 mmol/L. Demographic data, as well as data on lifestyle, dietary habits, animal exposure, and disease history were collected using a standardized questionnaire.

### Reporting summary

Further information on research design is available in the Nature Research Reporting Summary linked to this article.

## Supplementary information

Supplementary Information

Description of Additional Supplementary Files

Supplementary Data 1

Supplementary Data 2

Supplementary Data 3

Supplementary Data 4

Supplementary Data 5

Supplementary Data 6

Supplementary Data 7

Supplementary Data 8

Supplementary Data 9

Supplementary Data 10

Supplementary Data 11

Supplementary Data 12

Supplementary Data 13

Supplementary Data 14

Supplementary Data 15

Reporting Summary

## Data Availability

The stool metagenomic samples are available in the NCBI BioProject under accession number PRJNA686265. The mass spectrometry data for untargeted plasma metabolomics is accessible at http://www.ebi.ac.uk/metabolights/MTBLS2267. The data frame (R object) storing all the processed data in tabular format is available for download in the code repository specified below. Source data are provided with this paper.

## Source data

Source Data

## Code availability

Code to reproduce all the figures in this study is available at https://gitlab.com/xavier-lab-computation/public/fg300tanzania and 10.5281/zenodo.5015152.
