# Publisher Correction: Gut microbiome-mediated metabolism effects on immunity in rural and urban African populations

**DOI:** 10.1038/s41467-021-25472-z

**Published:** 2021-08-24

**Authors:** Martin Stražar, Godfrey S. Temba, Hera Vlamakis, Vesla I. Kullaya, Furaha Lyamuya, Blandina T. Mmbaga, Leo A. B. Joosten, Andre J. A. M. van der Ven, Mihai G. Netea, Quirijn de Mast, Ramnik J. Xavier

**Affiliations:** 1 Broad Institute of MIT and Harvard, Cambridge, MA USA; 2 Kilimanjaro Christian Medical University College, Moshi, Tanzania; 3 Kilimanjaro Clinical Research Institute, Kilimanjaro Christian Medical Centre, Moshi, Tanzania; 4 Department of Respiratory Medicine, Radboud University Medical Center, Nijmegen, The Netherlands; 5 Department of Pediatrics, Kilimanjaro Clinical Research Institute, Kilimanjaro Christian Medical Centre, Moshi, Tanzania; 6 Department of Internal Medicine, Radboud University Medical Center, Nijmegen, The Netherlands; 7 Center for Infectious Diseases, Radboud University Medical Center, Nijmegen, The Netherlands; 8 Center for Microbiome Informatics and Therapeutics, Massachusetts Institute of Technology, Cambridge, MA USA; 9 Center for Computational and Integrative Biology and Department of Molecular Biology, Massachusetts General Hospital and Harvard Medical School, Boston, MA USA

**Keywords:** Cytokines, Bacteria

Correction to: *Nature Communications* 10.1038/s41467-021-25213-2, published online 11 August 2021.

The original version of this Article incorrectly acknowledged Quirijn de Mast as a corresponding author instead of Ramnik J. Xavier. This has now been corrected in both the PDF and HTML versions of the Article.

