# Supplementary material for: Gut microbiome-mediated metabolism effects on immunity in rural and urban African populations
Source: Nat Commun. 2021 Aug 11;12:4845. doi: 10.1038/s41467-021-25213-2 (PMC8357928; doi:10.1038/s41467-021-25213-2)
Supplement: Supplementary file 1 — Supplementary Information [file 41467_2021_25213_MOESM1_ESM.pdf]

Gut microbiome-mediated metabolism effects on immunity in rural and urban populations

Supplementary Information

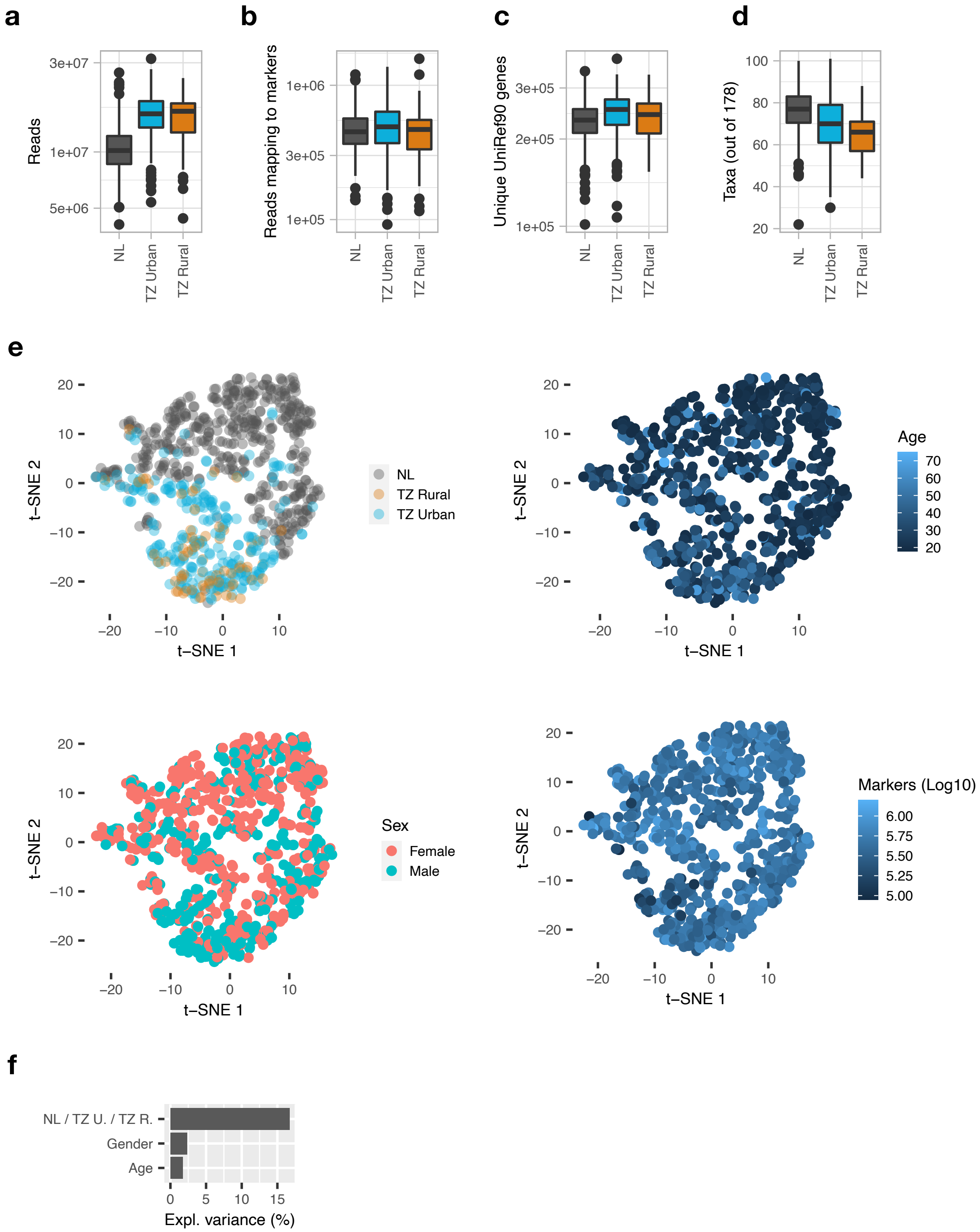

a
b
c
d
Reads
3e+07
1e+07
5e+06
Reads mapping to markers
1e+06
3e+05
1e+05
Unique UniRef90 genes
3e+05
2e+05
1e+05
Taxa (out of 178)
100
80
60
40
20
NL
TZ Urban
TZ Rural
e
t–SNE 1
t–SNE 2
NL
TZ Rural
TZ Urban
Age
70
60
50
40
30
20
Sex
Female
Male
Markers (Log10)
6.00
5.75
5.50
5.25
5.00
f
NL / TZ U. / TZ R.
Gender
Age
Expl. variance (%)
0
5
10
15

Supplementary Figure 1. Quality control and metagenomic profiling statistics between NL (n=471 biologically independent samples) TZ rural (n = 68) and TZ urban (n = 247) cohorts, with samples yielding at least 4 million reads. a) Number of reads after adapter and host DNA removal. The NL cohorts uses Illumina HiSeq 2000 platform (101 bp paired-end reads) while the TZ cohort was analyzed on Illumina NovaSeq 6000 (151bp paired-end). **b)** Reads mapping to marker genes in Metaphlan taxonomic profiling. **c)** Number of identified UniRef90 protein families. **d)** Number of detected species per subject among 176 species common to the two cohorts. Box plots show the median (center), first (Q1) and third quartile (Q3, box). Lower and upper whiskers represent Q1 + 1.5 IQR and Q3 + IQR, respectively. IQR, inter-quartile range. Data points outside whiskers‘ bounds are shown individually. **e)** t-SNE projection of microbial composition labeled by residency, sex, age and number of marker genes from the Metaphlan reference. **f)** PERMANOVA analysis of microbial compositions, represented by relative abundances of 176 common taxa. Bars show explained variance for residency, sex and age, all of which were significant at $P < 0.001$.

a

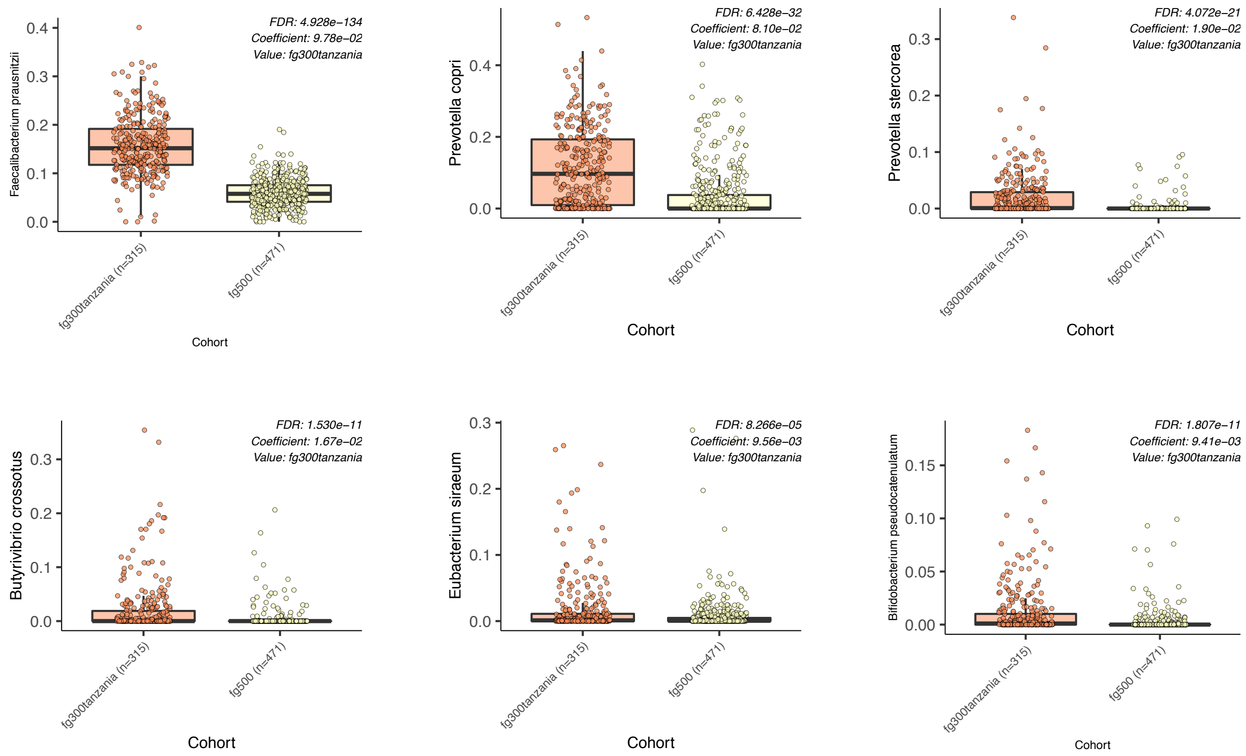
FDR: 4.928e−134
Coefficient: 9.78e−02
Value: fg300tanzania
Faecalibacterium prausnitzii
0.4
0.3
0.2
0.1
0.0
fg300tanzania (n=315)
fg500 (n=471)
Cohort
FDR: 6.428e−32
Coefficient: 8.10e−02
Value: fg300tanzania
Prevotella copri
0.4
0.2
0.0
fg300tanzania (n=315)
fg500 (n=471)
Cohort
FDR: 4.072e−21
Coefficient: 1.90e−02
Value: fg300tanzania
Prevotella stercorea
0.3
0.2
0.1
0.0
fg300tanzania (n=315)
fg500 (n=471)
Cohort
FDR: 1.530e−11
Coefficient: 1.67e−02
Value: fg300tanzania
Butyrivibrio crossotus
0.3
0.2
0.1
0.0
fg300tanzania (n=315)
fg500 (n=471)
Cohort
FDR: 8.266e−05
Coefficient: 9.56e−03
Value: fg300tanzania
Eubacterium siraeum
0.3
0.2
0.1
0.0
fg300tanzania (n=315)
fg500 (n=471)
Cohort
FDR: 1.807e−11
Coefficient: 9.41e−03
Value: fg300tanzania
Bifidobacterium pseudocatenulatum
0.15
0.10
0.05
0.00
fg300tanzania (n=315)
fg500 (n=471)
Cohort

b

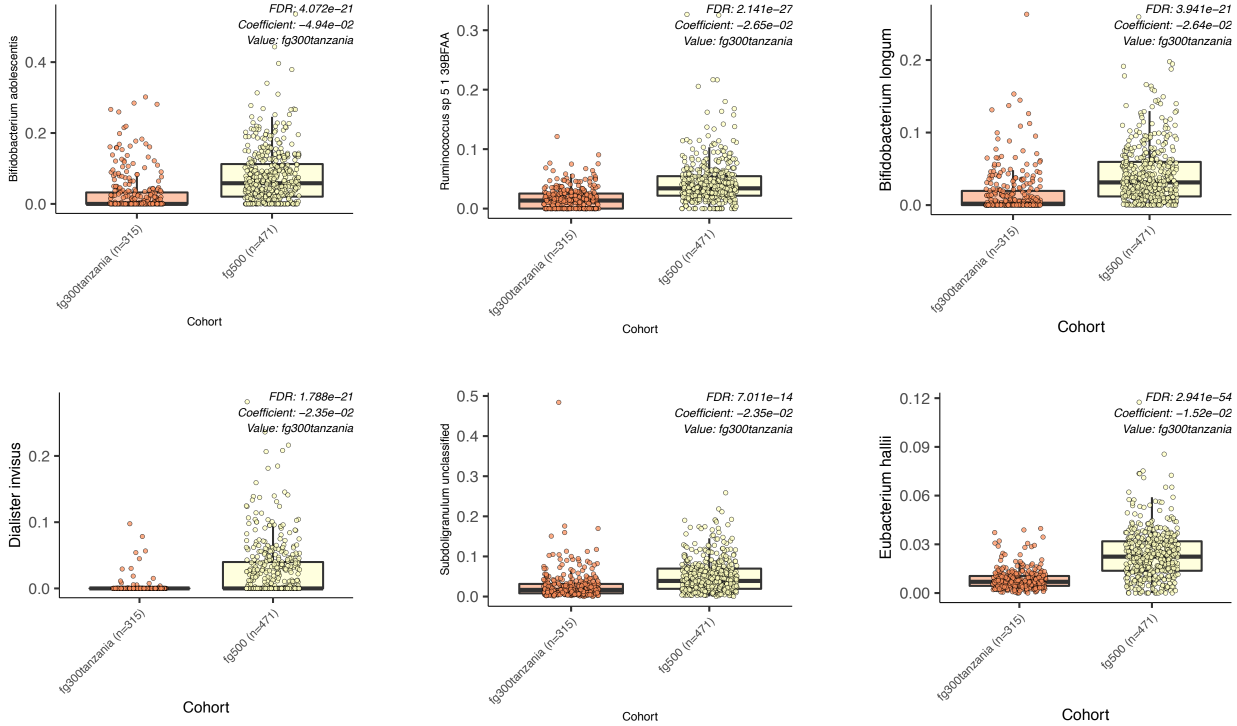
FDR: 4.072e−21
Coefficient: −4.94e−02
Value: fg300tanzania
Bifidobacterium adolescentis
0.4
0.2
0.0
fg300tanzania (n=315)
fg500 (n=471)
Cohort
FDR: 2.141e−27
Coefficient: −2.65e−02
Value: fg300tanzania
Ruminococcus sp 5 1 39BFAA
0.3
0.2
0.1
0.0
fg300tanzania (n=315)
fg500 (n=471)
Cohort
FDR: 3.941e−21
Coefficient: −2.64e−02
Value: fg300tanzania
Bifidobacterium longum
0.2
0.1
0.0
fg300tanzania (n=315)
fg500 (n=471)
Cohort
FDR: 1.788e−21
Coefficient: −2.35e−02
Value: fg300tanzania
Dialister invisus
0.2
0.1
0.0
fg300tanzania (n=315)
fg500 (n=471)
Cohort
FDR: 7.011e−14
Coefficient: −2.35e−02
Value: fg300tanzania
Subdoligranulum unclassified
0.5
0.4
0.3
0.2
0.1
0.0
fg300tanzania (n=315)
fg500 (n=471)
Cohort
FDR: 2.941e−54
Coefficient: −1.52e−02
Value: fg300tanzania
Eubacterium hallii
0.12
0.09
0.06
0.03
0.00
fg300tanzania (n=315)
fg500 (n=471)
Cohort

Supplementary Figure 2. Differences with between the Tanzanian (n = 315 biologically independent samples) and 500FG cohort (n = 471 biologically independent samples). The differential test is performed using Maaslin2 with cohort as a fixed effect and log-transformed relative abundance data. Out of total 104 significant species (FDR < 5%), the top six **a)** enriched and **b)** depleted species sorted by regression coefficient are displayed. Box plots show the median (center), first (Q1) and third quartile (Q3, box). Lower and upper whiskers represent Q1 + 1.5 IQR and Q3 + IQR, respectively. IQR, inter-quartile range. Data points outside whiskers' bounds are shown individually.

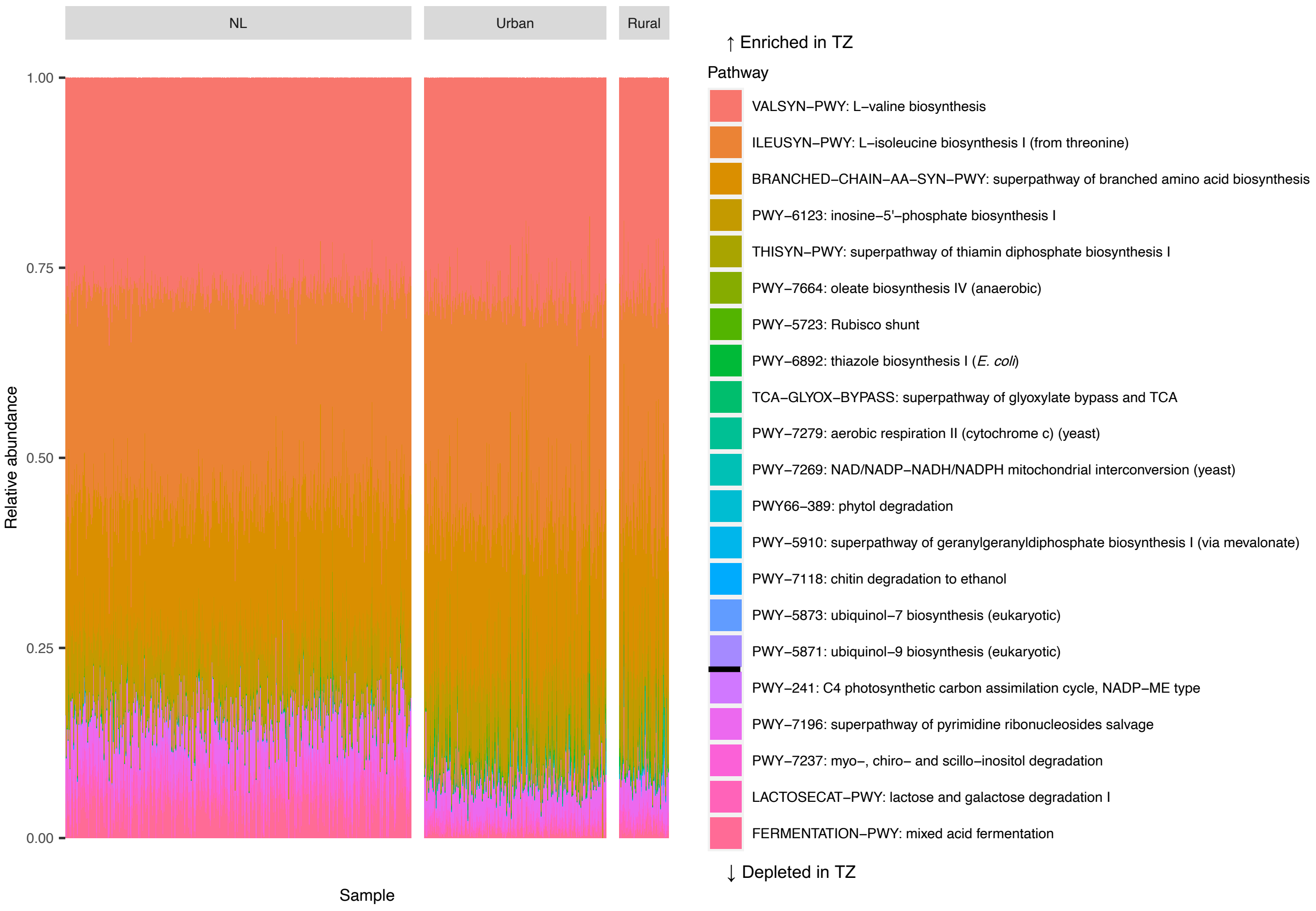
NL
Urban
Rural
1.00
0.75
0.50
0.25
0.00
Relative abundance
Sample
↑ Enriched in TZ
Pathway
VALSYN−PWY: L−valine biosynthesis
ILEUSYN−PWY: L−isoleucine biosynthesis I (from threonine)
BRANCHED−CHAIN−AA−SYN−PWY: superpathway of branched amino acid biosynthesis
PWY−6123: inosine−5'−phosphate biosynthesis I
THISYN−PWY: superpathway of thiamin diphosphate biosynthesis I
PWY−7664: oleate biosynthesis IV (anaerobic)
PWY−5723: Rubisco shunt
PWY−6892: thiazole biosynthesis I (E. coli)
TCA−GLYOX−BYPASS: superpathway of glyoxylate bypass and TCA
PWY−7279: aerobic respiration II (cytochrome c) (yeast)
PWY−7269: NAD/NADP−NADH/NADPH mitochondrial interconversion (yeast)
PWY66−389: phytol degradation
PWY−5910: superpathway of geranylgeranyldiphosphate biosynthesis I (via mevalonate)
PWY−7118: chitin degradation to ethanol
PWY−5873: ubiquinol−7 biosynthesis (eukaryotic)
PWY−5871: ubiquinol−9 biosynthesis (eukaryotic)
PWY−241: C4 photosynthetic carbon assimilation cycle, NADP−ME type
PWY−7196: superpathway of pyrimidine ribonucleosides salvage
PWY−7237: myo−, chiro− and scillo−inositol degradation
LACTOSECAT−PWY: lactose and galactose degradation I
FERMENTATION−PWY: mixed acid fermentation
↓ Depleted in TZ

Supplementary Figure 3. Functional diversity in metagenomic pathways between cohorts. Reads mapping to MetaCyc pathways were quantified using patway copy numbers using Humann2. The differential relative abundance of pathways was analyzed using Maaslin2, with residency (NL, TZ urban, TZ rural), age and sex as fixed effects and cohort (NL or TZ) as a random effect. Differentially abundant pathways were selected subject to FDR < 0.05 and presence in at least 20 samples. The bar plots show relative abundance within 21 selected pathways for each subject. Black line in the legend separates pathways enriched in TZ (16) and those enriched in NL (5).

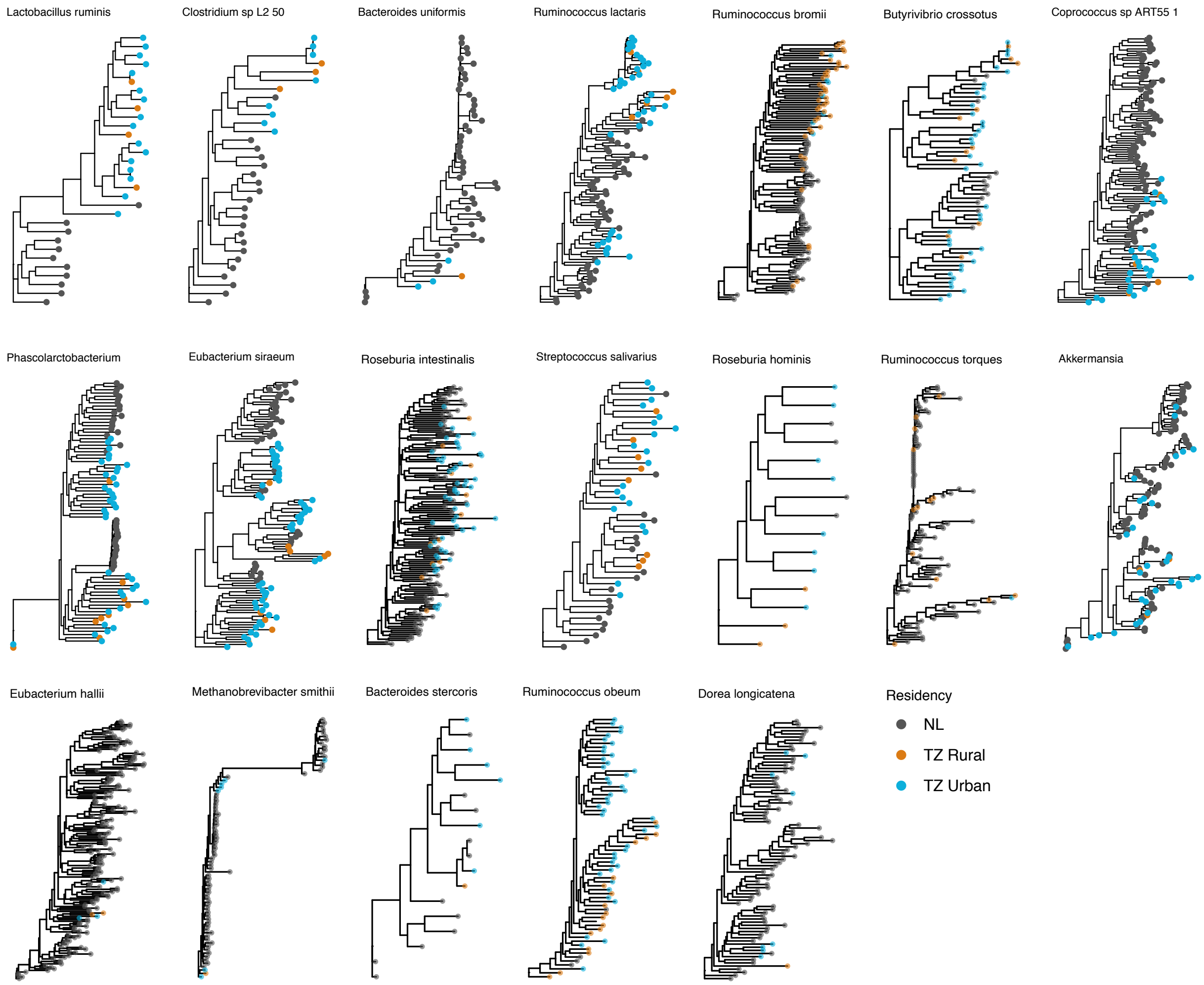
Lactobacillus ruminis
Clostridium sp L2 50
Bacteroides uniformis
Ruminococcus lactaris
Ruminococcus bromii
Butyrivibrio crossotus
Coprococcus sp ART55 1
Phascolarctobacterium
Eubacterium siraeum
Roseburia intestinalis
Streptococcus salivarius
Roseburia hominis
Ruminococcus torques
Akkermansia
Eubacterium hallii
Methanobrevibacter smithii
Bacteroides stercoris
Ruminococcus obeum
Dorea longicatena
Residency
NL
TZ Rural
TZ Urban

Supplementary Figure 4. Strain analysis of NL, TZ Urban and TZ Rural cohorts with Strainphlan. Significance strain divergence driven by residency for each taxon was estimated using the PERMANOVA analysis using the underlying phylogenetic distances matrices ($P < 0.05$, minimum samples = 10, presence in all three residency areas).

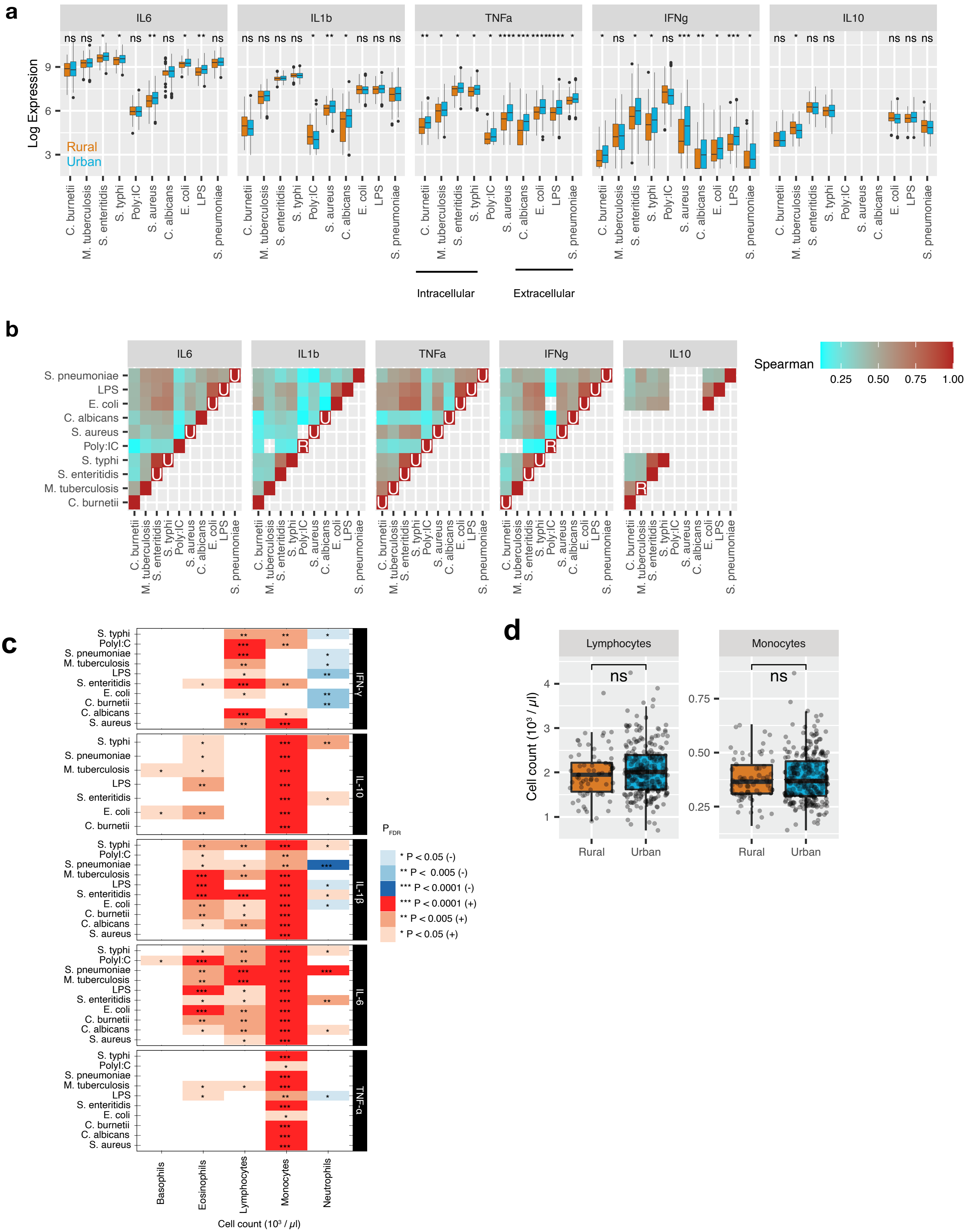
a
IL6
IL1b
TNFa
IFNg
IL10
Log Expression
Rural
Urban
C. burnetii
M. tuberculosis
S. enteritidis
S. typhi
Poly:IC
S. aureus
C. albicans
E. coli
LPS
S. pneumoniae
Intracellular
Extracellular
b
Spearman
0.25
0.50
0.75
1.00
c
IFN-γ
IL-10
IL-1β
IL-6
TNF-α
S. typhi
PolyI:C
S. pneumoniae
M. tuberculosis
LPS
S. enteritidis
E. coli
C. burnetii
C. albicans
S. aureus
Basophils
Eosinophils
Lymphocytes
Monocytes
Neutrophils
Cell count (10³ / μl)
P FDR
* P < 0.05 (-)
** P < 0.005 (-)
*** P < 0.0001 (-)
*** P < 0.0001 (+)
** P < 0.005 (+)
* P < 0.05 (+)
d
Lymphocytes
Monocytes
Cell count (10³ / μl)
ns
Rural
Urban

Supplementary Figure 5. Cytokine expression in rural and urban TZ cohorts. a) Box plots of cytokine expression levels upon ex vivo stimulations for TZ rural (n=68) and TZ urban (n=239) cohorts. The difference between urban and rural sample levels is assessed by two-sided Wilcoxon Rank Sum test (*P <= 0.05, **P <= 0.01, ***P <= 0.001, ****P <= 0.0001). Box plots show the median (center), first (Q1) and third quartile (Q3, box). Lower and upper whiskers represent Q1 + 1.5 IQR and Q3 + IQR, respectively. IQR, inter-quartile range. Data points outside whiskers‘ bounds are shown individually. **b)** Significant Spearman correlations (P< 0.05) between samples in cytokine responses to all pairs of stimuli. Diagonal entries display significantly increased levels in urban (U) or rural (R) samples (Wilcoxon Rank Sum test, P < 0.05). **c)** Spearman correlation between counts of basophils, lymphocytes monocytes and neutrophils versus all measured cytokine responses. Blue and red respectively denote significant negative and positive correlation coefficients after FDR correction. **d)** Comparison of relative monocyte and lymphocyte counts (103 / µl) in urban (n=239) and rural (n=68) whole blood samples. Definition of box plot as in a. Significance assessed with two-sided Wilcoxon Rank Sum test.

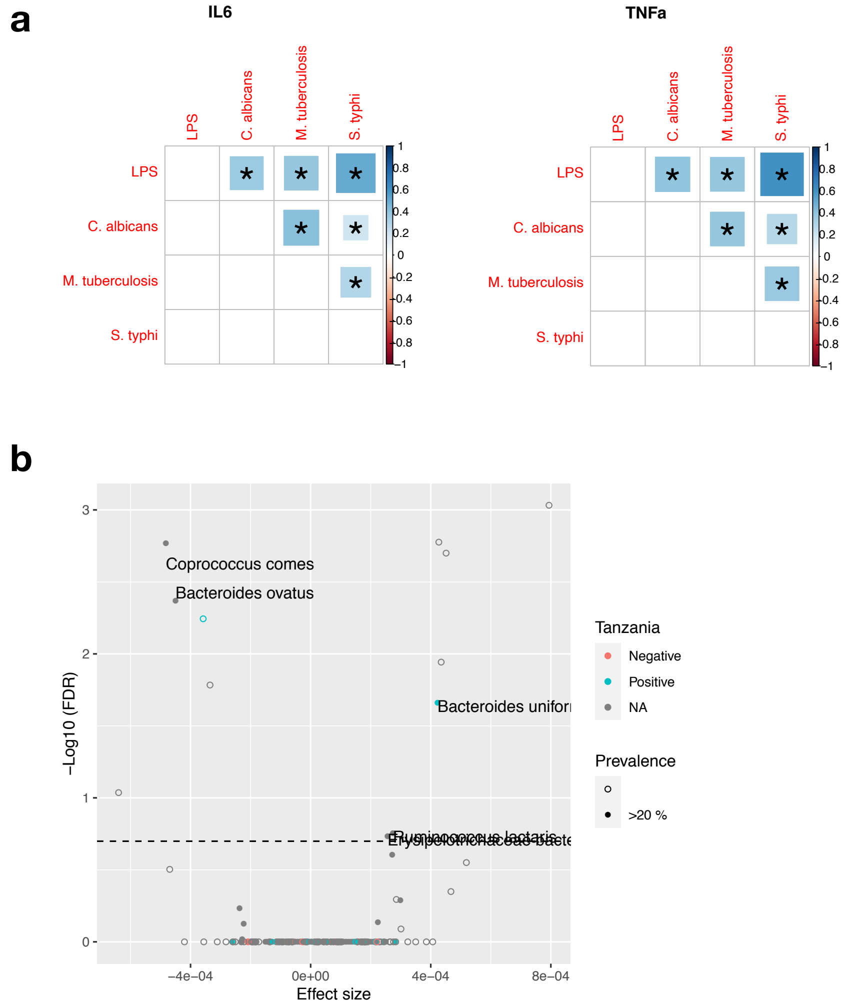
a
IL6
TNFa
LPS
C. albicans
M. tuberculosis
S. typhi
*
1
0.8
0.6
0.4
0.2
0
-0.2
-0.4
-0.6
-0.8
-1
b
Coprococcus comes
Bacteroides ovatus
Bacteroides unifor
Ruminococcus lactaris
Erysipelotrichaceae bacte
-Log10 (FDR)
Effect size
-4e-04
0e+00
4e-04
8e-04
Tanzania
Negative
Positive
NA
Prevalence
>20 %

Supplementary Figure 6. Overlapping cytokine responses in the 500 FG cohort. a) Spearman correlation plots between cytokine responses for different stimuli. **b)** Log-linear model of immunomodulatory applied to the 500FG cohort (same as with TZ cohort; Methods).

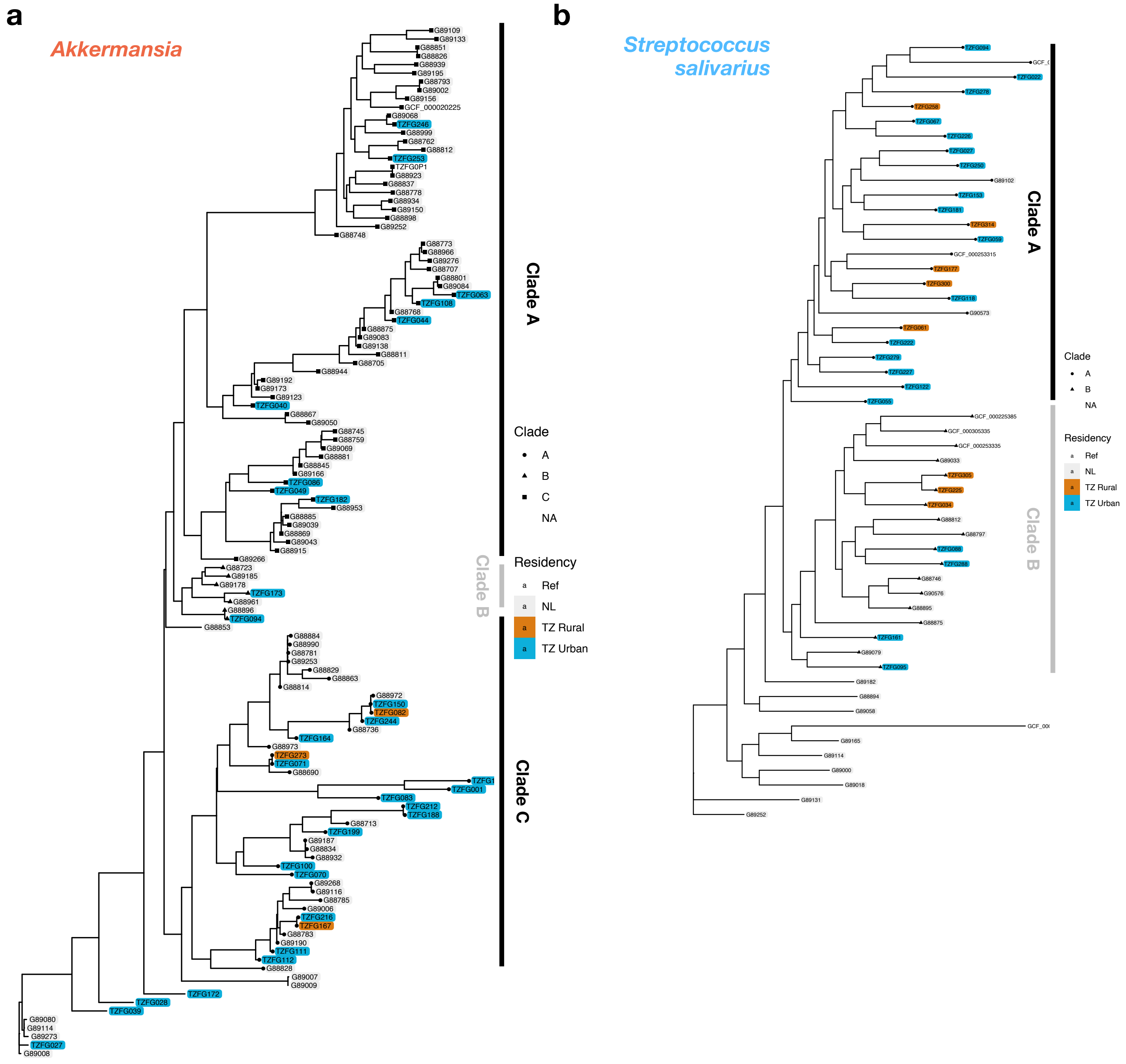

c

	effect	p-value
Residency_Area Urban	-0.03	0.606
Akkermansia Clade B	0.11	0.183
***Akkermansia* Clade C**	**0.08**	**0.070 .**

d

	effect	pvalue
Residency Area Urban	0.262	3.28e-08 ***
S. salivarius Clade B	0.262	1.13e-07 ***

Supplementary Figure 7. Clade analysis for two immunomodulatory taxa, *Akkermansia* and *S. Salivarius*. a-b) Phylogenetic trees of samples from urban, rural (TZ) and Dutch samples obtained with StrainPhlan. Clades are determined such that the ordering within them retains a significant split in rural-urban-TZ (Kruskal-Wallis test). **c-d)** Clade effects on cytokine responses using the ANOVA model of the form Log Cytokine Expression ~ Age + Gender + Residency + Clade, where the observation including the taxa are used.

a

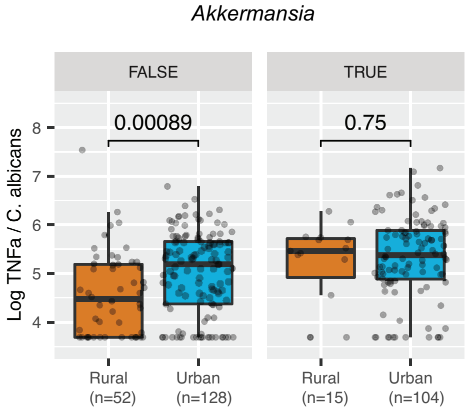

Akkermansia
FALSE
TRUE
0.00089
0.75
Log TNFa / C. albicans
8
7
6
5
4
Rural
(n=52)
Urban
(n=128)
Rural
(n=15)
Urban
(n=104)

b

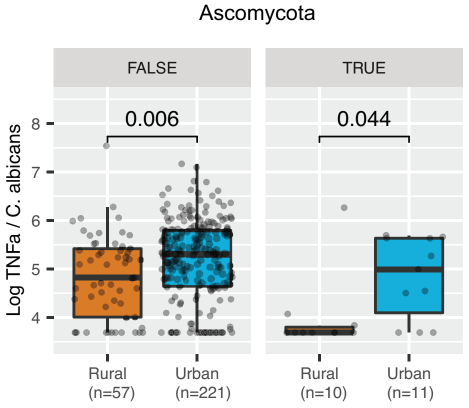

Ascomycota
FALSE
TRUE
0.006
0.044
Log TNFa / C. albicans
8
7
6
5
4
Rural
(n=57)
Urban
(n=221)
Rural
(n=10)
Urban
(n=11)

Supplementary Figure 8. Effect on expression of TNF-α stimulated with *C. albicans* in rural and urban samples. Expression based on detection of **a)** Akkermansia and **b)** Ascomycota (two-tailed Wilcoxon rank sum test, n denotes the number of biologically independent samples in each category). Box plots show the median (center), first (Q1) and third quartile (Q3, box). Lower and upper whiskers represent Q1 + 1.5 IQR and Q3 + IQR, respectively. IQR, inter-quartile range. Data points outside whiskers‘ bounds are shown individually.

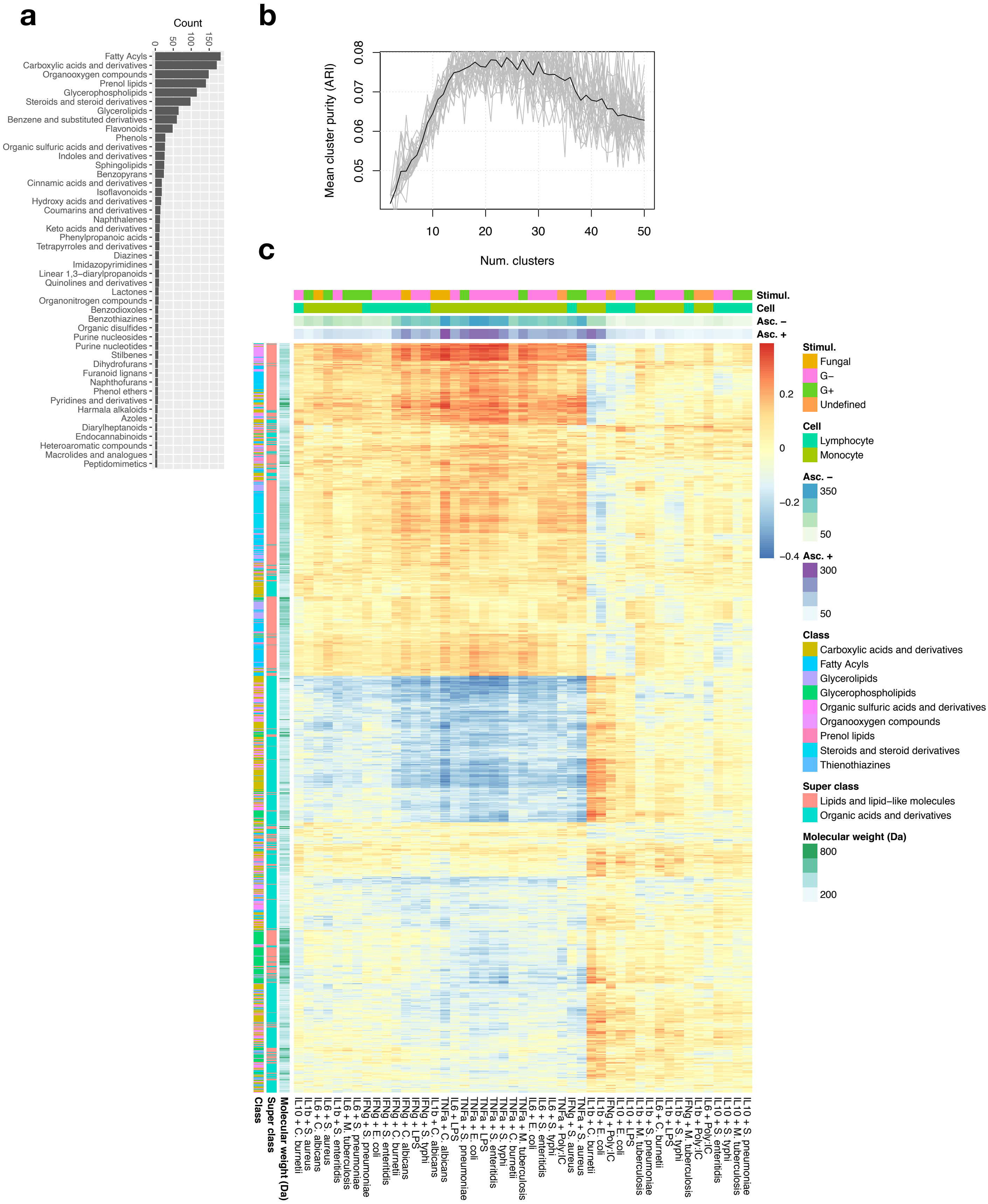

a
Count
0
50
100
150
Fatty Acyls
Carboxylic acids and derivatives
Organooxygen compounds
Prenol lipids
Glycerophospholipids
Steroids and steroid derivatives
Glycerolipids
Benzene and substituted derivatives
Flavonoids
Phenols
Organic sulfuric acids and derivatives
Indoles and derivatives
Sphingolipids
Benzopyrans
Cinnamic acids and derivatives
Isoflavonoids
Hydroxy acids and derivatives
Coumarins and derivatives
Naphthalenes
Keto acids and derivatives
Phenylpropanoic acids
Tetrapyrroles and derivatives
Diazines
Imidazopyrimidines
Linear 1,3−diarylpropanoids
Quinolines and derivatives
Lactones
Organonitrogen compounds
Benzodioxoles
Benzothiazines
Organic disulfides
Purine nucleosides
Purine nucleotides
Stilbenes
Dihydrofurans
Furanoid lignans
Naphthofurans
Phenol ethers
Pyridines and derivatives
Harmala alkaloids
Azoles
Diarylheptanoids
Endocannabinoids
Heteroaromatic compounds
Macrolides and analogues
Peptidomimetics
b
Mean cluster purity (ARI)
0.05
0.06
0.07
0.08
10
20
30
40
50
Num. clusters
c
Stimul.
Cell
Asc. –
Asc. +
0.2
0
−0.2
−0.4
Stimul.
Fungal
G−
G+
Undefined
Cell
Lymphocyte
Monocyte
Asc. –
350
50
Asc. +
300
50
Class
Carboxylic acids and derivatives
Fatty Acyls
Glycerolipids
Glycerophospholipids
Organic sulfuric acids and derivatives
Organooxygen compounds
Prenol lipids
Steroids and steroid derivatives
Thienothiazines
Super class
Lipids and lipid−like molecules
Organic acids and derivatives
Molecular weight (Da)
800
200
Class
Super class
Molecular weight (Da)
IL10 + C. burnetii
IL1b + S. aureus
IL6 + C. albicans
IL6 + S. aureus
IL1b + S. enteritidis
IL6 + M. tuberculosis
IL6 + S. pneumoniae
IFNg + S. pneumoniae
IFNg + E. coli
IFNg + S. enteritidis
IFNg + C. burnetii
IFNg + C. albicans
IFNg + LPS
IFNg + S. typhi
IL1b + C. albicans
TNFa + C. albicans
IL6 + LPS
TNFa + S. pneumoniae
TNFa + E. coli
TNFa + LPS
TNFa + S. enteritidis
TNFa + S. typhi
TNFa + C. burnetii
TNFa + M. tuberculosis
IL6 + E. coli
IL6 + S. enteritidis
IL6 + S. typhi
TNFa + Poly:IC
IFNg + S. aureus
TNFa + S. aureus
IL1b + C. burnetii
IL1b + E. coli
IFNg + Poly:IC
IL10 + E. coli
IL10 + LPS
IL1b + M. tuberculosis
IL1b + S. pneumoniae
IL6 + C. burnetii
IL1b + LPS
IL1b + S. typhi
IFNg + M. tuberculosis
IL1b + Poly:IC
IL6 + Poly:IC
IL10 + S. enteritidis
IL10 + S. typhi
IL10 + M. tuberculosis
IL10 + S. pneumoniae

Supplementary Figure 9. Clustering and quality control of serum metabolomics data. A total of 1,607 m/z peaks identified by MS/MS were identified and matched to at least one molecule in the Human Metabolome Database (HMDB) **a)** The distribution of molecular classes for measured molecules. **b)** Number of clusters (28) is selected such that the clusters show maximal correspondence with the molecular classes as measured by the Adjusted Random Index (ARI). The clustering is repeated 50 times for each number of clusters of K with different random initializations. **c)** Significant spearman correlations between cytokine responses and metabolites (Spearman correlation, PFDR < 0.05).

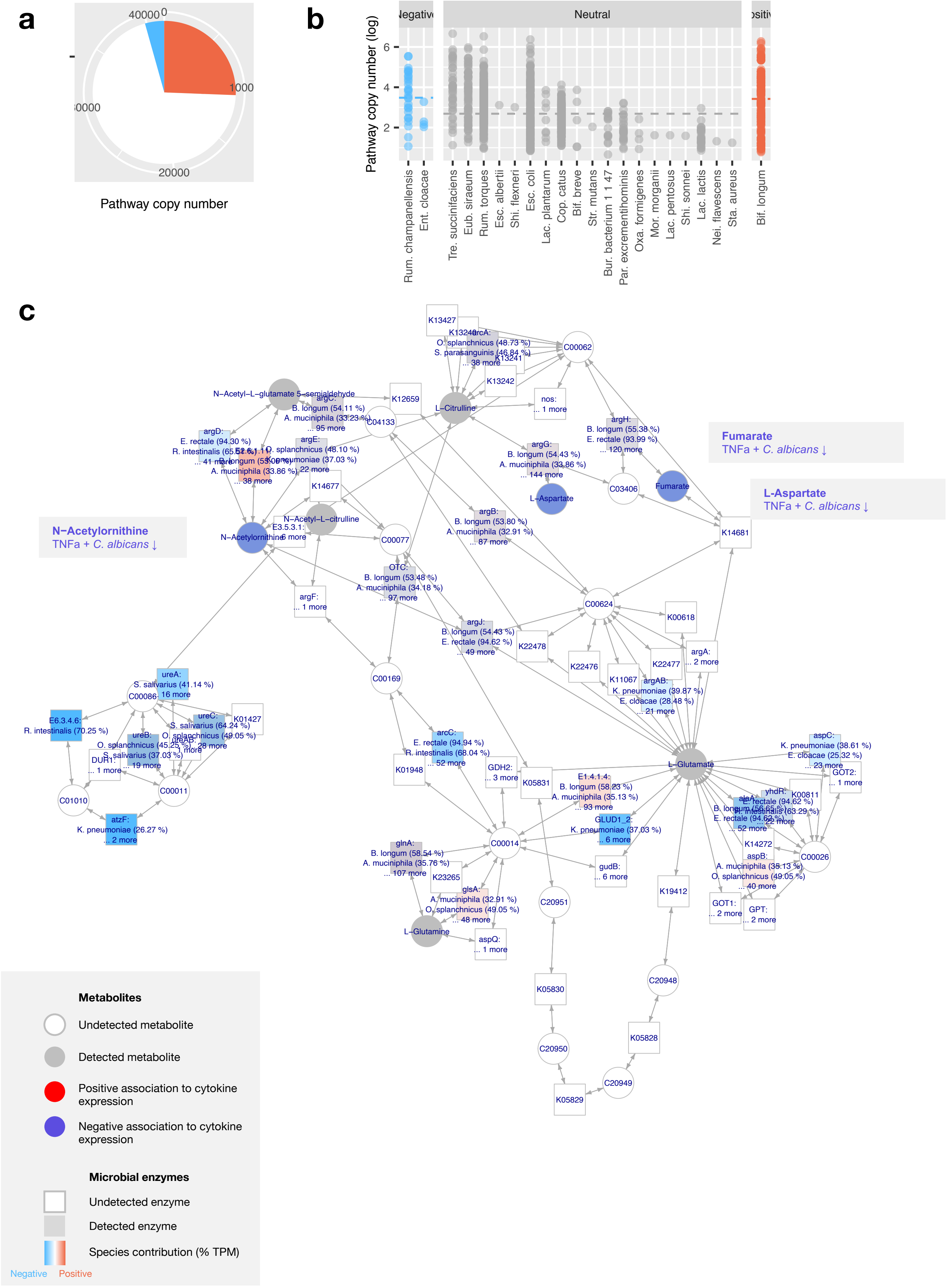
a
b
c
Pathway copy number
40000
0
1000
20000
Pathway copy number (log)
Negative
Neutral
Rum. champanellensis
Ent. cloacae
Tre. succinifaciens
Eub. siraeum
Rum. torques
Esc. albertii
Shi. flexneri
Esc. coli
Lac. plantarum
Cop. catus
Bif. breve
Str. mutans
Bur. bacterium 1 1 47
Par. excrementihominis
Oxa. formigenes
Mor. morganii
Lac. pentosus
Shi. sonnei
Lac. lactis
Nei. flavescens
Sta. aureus
Bif. longum
Fumarate
TNFa + C. albicans ↓
L-Aspartate
TNFa + C. albicans ↓
N−Acetylornithine
TNFa + C. albicans ↓
L-Citrulline
L-Glutamate
L-Glutamine
N−Acetylornithine
N−Acetyl−L−citrulline
N−Acetyl−L−glutamate 5−semialdehyde
Metabolites
Undetected metabolite
Detected metabolite
Positive association to cytokine expression
Negative association to cytokine expression
Microbial enzymes
Undetected enzyme
Detected enzyme
Species contribution (% TPM)
Negative
Positive

Supplementary Figure 10. Arginine biosynthesis pathway detection in microbial species. a, b) Copy numbers of arginine biosynthesis pathway II (MetaCyc, ARGSYNBSUB), detected in the metagenome of negative, neutral and positive species. **c)** Detected enzymes, metabolites and associations in arginine biosynthesis pathway (KEGG KO00220). Colored nodes represent detected microbial enzymes (squares) and compounds (circles), with significant correlations with cytokine responses in red (positive) and blue (negative). Enzymes detected in stool microbiomes are shown as colored squares, with contribution from negative (blue) or positive species (red) quantified as fraction of total RPKM in the cohort.
