# Supplementary material for: Gut microbiome-mediated metabolism effects on immunity in rural and urban African populations
Source: Nat Commun. 2021 Aug 11;12:4845. doi: 10.1038/s41467-021-25213-2 (PMC8357928; doi:10.1038/s41467-021-25213-2)
Supplement: Supplementary file 2 — Description of Additional Supplementary Files [file 41467_2021_25213_MOESM2_ESM.docx]

**Supplementary Information**

**Description of Additional Supplementary Files**

File Name: Supplementary Data 1

Description: Health-related and socio-economic information per subject.

File Name: Supplementary Data 2

Description: Survey of dietary information.

File Name: Supplementary Data 3

Description: Microbial species changing along gradients rural-urban-NL (RUN), urban-rural-NL (URN) and rural-NL-urban (RNU). Significance values obtained by Wilcoxon one-sided tests and FDR multiple testing adjustment.

File Name: Supplementary Data 4

Description: Microbial species enrichment in urban, rural and Dutch samples . Significance values obtained by Maaslin2 (two-sided T-test and FDR adjustment)

File Name: Supplementary Data 5

Description: Taxa with significant strain-level divergence between urban, rural and Dutch samples (StrainPhlan).

File Name: Supplementary Data 6

Description: Changes in phyla abundance with selected variables using Maaslin2 (two-tailed T-test and FDR adjustment).

File Name: Supplementary Data 7

Description: Chi-Square test was used for categorical variables (Meta type, intestine, alcohol type) and Kruskall-Wallis test was used for variables measured as weekly frequency (wheat, vegetables, ugali, sweet tea, rice, potato chips, milk, meat, fruits, fish

carbonated soda, beans, banana). Significance values were corrected and FDR multiple testing adjustment.

File Name: Supplementary Data 8

Description: Correlations between cytokine expressions, stimuli and residency area. Spearman correlation tests and FDR multiple testing adjustment were used to measure correlation between expressions of different cytokines and stimuli. Wilcoxon two-sided tests and FDR multiple testing adjustment was used to compare differences in cytokine expressions among urban and rural individuals.

File Name: Supplementary Data 9

Description: Immunomodulatory species identified by the log-linear model. Significance values obtained by two-sided T-test and Bonferroni adjustment.

File Name: Supplementary Data10

Description: Species with significant explained variance with respect to at least one cytokine response and stimulus, corrected for age and sex (Pearson correlation test, unadjusted).

File Name: Supplementary Data 11

Description: Differentially abundant metabolites between urban and rural samples. Wilcoxon Rank Sum two sided test with Bonferroni adjustment.

File Name: Supplementary Data 12

Description: Detected compounds mapped to KEGG Compound identifiers and related metabolic pathways.

File Name: Supplementary Data 13

Description: Spearman correlation between metabolite intensity and cytokine expressions. Significance values obtained by two-sided Spearman correlation tests and FDR multiple testing adjustment.

File Name: Supplementary Data 14

Description: Spearman correlation between metabolite intensity and microbial species abundance. Significance values obtained by two-sided Spearman correlation tests and FDR multiple testing adjustment.

File Name: Supplementary Data 15

Description: Pathway copy number enrichment in negative and positive immunomodulatory species. Significance values obtained by the log-linear model (unadjusted p-values, two-sided T-test).
