# Supplementary material for: Gut microbiome-mediated metabolism effects on immunity in rural and urban African populations
Source: Nat Commun. 2021 Aug 11;12:4845. doi: 10.1038/s41467-021-25213-2 (PMC8357928; doi:10.1038/s41467-021-25213-2)
Supplement: Supplementary file 18 — Reporting Summary [file 41467_2021_25213_MOESM18_ESM.pdf]

nature research

Corresponding author(s): Ramnik J. Xavier
Last updated by author(s): Jan 8, 2021

Reporting Summary

Nature Research wishes to improve the reproducibility of the work that we publish. This form provides structure for consistency and transparency in reporting. For further information on Nature Research policies, see our Editorial Policies and the Editorial Policy Checklist.

Statistics

For all statistical analyses, confirm that the following items are present in the figure legend, table legend, main text, or Methods section.

n/a	Confirmed	
☐	☒	The exact sample size (n) for each experimental group/condition, given as a discrete number and unit of measurement
☐	☒	A statement on whether measurements were taken from distinct samples or whether the same sample was measured repeatedly
☐	☒	The statistical test(s) used AND whether they are one- or two-sided<br>*Only common tests should be described solely by name; describe more complex techniques in the Methods section.*
☐	☒	A description of all covariates tested
☐	☒	A description of any assumptions or corrections, such as tests of normality and adjustment for multiple comparisons
☐	☒	A full description of the statistical parameters including central tendency (e.g. means) or other basic estimates (e.g. regression coefficient) AND variation (e.g. standard deviation) or associated estimates of uncertainty (e.g. confidence intervals)
☐	☒	For null hypothesis testing, the test statistic (e.g. F, t, r) with confidence intervals, effect sizes, degrees of freedom and P value noted<br>*Give P values as exact values whenever suitable.*
☒	☐	For Bayesian analysis, information on the choice of priors and Markov chain Monte Carlo settings
☒	☐	For hierarchical and complex designs, identification of the appropriate level for tests and full reporting of outcomes
☐	☒	Estimates of effect sizes (e.g. Cohen's d, Pearson's r), indicating how they were calculated

Our web collection on statistics for biologists contains articles on many of the points above.

Software and code

Policy information about availability of computer code

Data collection	Data were collected using cloud-based electronic data capture (Castor EDC 2017.2 EU server)
Data analysis	Metabolome data was processed using Matlab R2018a. Metagenomics was processed using the biobakery pipeline (https://huttenhower.sph.harvard.edu/biobakery_workflows/), based on MetaPhlAn 2.2. The R code to reproduce the analyses and a data object storing all the required data are available at https://gitlab.com/xavier-lab-computation/public/fg300tanzania. The versions of R packages are as follows: vegan_2.5-7, lattice_0.20-38, permute_0.9-5, stringr_1.4.0, scales_1.0.0, reshape2_1.4.3, pvclust_2.2-0, philentropy_0.4.0, pheatmap_1.0.12, mltools_0.3.5, mclust_5.4.5, , igraph_1.2.4.1, ggtree_2.0.4, ggrepel_0.8.2, ggpubr_0.2.4, , magrittr_1.5, fmsb_0.7.0, , entropy_1.2.1, egg_0.4.5, ggplot2_3.3.0, gridExtra_2.3, data.table_1.12.8, corrplot_0.84, colorspace_1.4-1, ape_5.4-1, , Rtsne_0.15, Maaslin2_1.0.0, CCA_1.2, fields_10.0, maps_3.3.0, spam_2.4-0, dotCall64_1.0-0, fda_2.4.8, Matrix_1.2-17.

For manuscripts utilizing custom algorithms or software that are central to the research but not yet described in published literature, software must be made available to editors and reviewers. We strongly encourage code deposition in a community repository (e.g. GitHub). See the Nature Research guidelines for submitting code & software for further information.

Data

Policy information about availability of data

All manuscripts must include a data availability statement. This statement should provide the following information, where applicable:

- Accession codes, unique identifiers, or web links for publicly available datasets
- A list of figures that have associated raw data
- A description of any restrictions on data availability

The data that support the findings of this study are available from the corresponding author upon request. Raw metagenomic reads were deposited to NCBI BioProject under accession PRJNA686265. Metabolomics data has been deposited to the EMBL-EBI MetaboLights database 65 with study identifier MTBLS2267. The

complete data can be accessed at: www.ebi.ac.uk/metabolights/MTBLS2267.

Field-specific reporting

Please select the one below that is the best fit for your research. If you are not sure, read the appropriate sections before making your selection.

☒ Life sciences ☐ Behavioural & social sciences ☐ Ecological, evolutionary & environmental sciences

For a reference copy of the document with all sections, see nature.com/documents/nr-reporting-summary-flat.pdf

Life sciences study design

All studies must disclose on these points even when the disclosure is negative.

Sample size	In earlier studies by our collaboration group, the associations between genome and immunity have been reliable obtained in a group of approximately 100 volunteers (Smeekens et al, Nature Communications 2013). Because we intend to add an additional aspect to this analysis, and assess the interaction between metagenome, metabolome and immunity we decided to increase the number of individuals tested to 300.<br><br>We report on standard errors resulting from a finite sample size and adjusted significant values due to multiple hypothesis testing with all the models applied to the metabolomics and metagenomics and immunological data.
Data exclusions	Metagenomic samples were excluded if they contained less than 4 million reads (n=8) as a part of the pre-established quality control criterion. These were removed in experiments in Fig. 2-Fig.6, and Suppl. Fig. 1-Suppl.Fig 11
Replication	The replicability of findings was successfully replicated in three following aspects, each of which were performed once.<br><br>1) in an independent cohort with similar design (500 FG, Netherlands), which used a smaller set of cytokine readouts. The effects of immunomodulatory microbes were nevertheless significantly correlated.<br><br>3) Immunomodulatory species derived from correlations with cytokine expression also showed larger effects on independently measured circulating metabolites.<br><br>2) The importance of metabolic pathways was confirmed through independently measured circulating metabolites and metagenomics, using two different annotation systems (KEGG and MetaCyc).
Randomization	No randomization was required since this study was designed as a cross sectional study in which healthy volunteers from rural and urban areas were sampled once without a follow up. All participants were coded anonymously and all downstream measurements and statistical analyses used only these codes. Covariates like age and sex were controlled for during analyses.
Blinding	Blinding was not relevant in this type of study design but all participants were coded anonymously and all downstream measurements and statistical analyses used only these codes.

Reporting for specific materials, systems and methods

We require information from authors about some types of materials, experimental systems and methods used in many studies. Here, indicate whether each material, system or method listed is relevant to your study. If you are not sure if a list item applies to your research, read the appropriate section before selecting a response.

Materials & experimental systems

n/a	Involved in the study	
☒	☐	Antibodies
☒	☐	Eukaryotic cell lines
☒	☐	Palaeontology and archaeology
☒	☐	Animals and other organisms
☐	☒	Human research participants
☒	☐	Clinical data
☒	☐	Dual use research of concern

Methods

n/a	Involved in the study	
☒	☐	ChIP-seq
☒	☐	Flow cytometry
☒	☐	MRI-based neuroimaging

Human research participants

Policy information about studies involving human research participants

Population characteristics	A total of 323 Tanzanian healthy individuals aged between 18-65 years residing within the Kilimanjaro region in Northern Tanzania were enrolled between March and December 2017, at the Kilimanjaro Christian Medical Center and Lucy Lameck Research Center, in Moshi municipal. Participants from both rural and urban areas were enrolled. Approx. 50% males and females were recruited. Exclusion criteria were pregnancy, a known acute or chronic disease, use of antibiotics or anti-malarials in the previous three months, or receiving treatment for tuberculosis infection in the past year.
Recruitment	Information on the study was given through leaflets or announced during the mass gathering. All volunteers were interviewed by a member of the study team using a guided pre-screening questionnaire prior to being invited to the study center
Ethics oversight	Ethical Committees of the Kilimanjaro Christian Medical University College (CREC) (No 2443) and the National Institute for Medical Research in Tanzania (NIMR/HQ/R.8a/Vol. IX/2290 and NIMR/HQ/R.8a/Vol.IX/3318). In the Netherlands, the study was approved by the Ethical Committee of the Radboud University Medical Centre Nijmegen (CMO Arnhem-Nijmegen; 2016-2657).

Note that full information on the approval of the study protocol must also be provided in the manuscript.
